# Mitigation of Ionizing Radiation-Induced Gastrointestinal Damage by Insulin-Like Growth Factor-1 in Mice

**DOI:** 10.3389/fphar.2022.663855

**Published:** 2022-06-29

**Authors:** Jaroslav Pejchal, Ales Tichy, Adela Kmochova, Lenka Fikejzlova, Klara Kubelkova, Marcela Milanova, Anna Lierova, Alzbeta Filipova, Lubica Muckova, Jana Cizkova

**Affiliations:** ^1^ Department of Toxicology and Military Pharmacy, Faculty of Military Health Sciences, University of Defence, Brno, Czechia; ^2^ Department of Radiobiology, Faculty of Military Health Sciences, University of Defence, Brno, Czechia; ^3^ Department of Molecular Pathology and Biology, Faculty of Military Health Sciences, University of Defence, Brno, Czechia

**Keywords:** ionizing radiation, mice, insuline-like growth factor- 1, intestine, lung, blood

## Abstract

**Purpose:** Insulin-like growth factor-1 (IGF-1) stimulates epithelial regeneration but may also induce life-threatening hypoglycemia. In our study, we first assessed its safety. Subsequently, we examined the effect of IGF-1 administered in different dose regimens on gastrointestinal damage induced by high doses of gamma radiation.

**Material and methods:** First, fasting C57BL/6 mice were injected subcutaneously with IGF-1 at a single dose of 0, 0.2, 1, and 2 mg/kg to determine the maximum tolerated dose (MTD). The glycemic effect of MTD (1 mg/kg) was additionally tested in non-fasting animals. Subsequently, a survival experiment was performed. Animals were irradiated (^60^Co; 14, 14.5, or 15 Gy; shielded head), and IGF-1 was administered subcutaneously at 1 mg/kg 1, 24, and 48 h after irradiation. Simultaneously, mice were irradiated (^60^Co; 12, 14, or 15 Gy; shielded head), and IGF-1 was administered subcutaneously under the same regimen. Jejunum and lung damage were assessed 84 h after irradiation. Finally, we evaluated the effect of six different IGF-1 dosage regimens administered subcutaneously on gastrointestinal damage and peripheral blood changes in mice 6 days after irradiation (^60^Co; 12 and 14 Gy; shielded head). The regimens differed in the number of doses (one to five doses) and the onset of administration (starting at 1 [five regimens] or 24 h [one regimen] after irradiation).

**Results:** MTD was established at 1 mg/kg. MTD mitigated lethality induced by 14 Gy and reduced jejunum and lung damage caused by 12 and 14 Gy. However, different dosing regimens showed different efficacy, with three and four doses (administered 1, 24, and 48 h and 1, 24, 48, and 72 h after irradiation, respectively) being the most effective. The three-dose regimens supported intestinal regeneration even if the administration started at 24 h after irradiation, but its potency decreased.

**Conclusion:** IGF-1 seems promising in the mitigation of high-dose irradiation damage. However, the selected dosage regimen affects its efficacy.

## 1 Introduction

Acute gastrointestinal radiation syndrome (GRS) is a life-threatening situation that develops after exposure of the gastrointestinal tract to high doses of ionizing radiation (IR). The pathogenesis of acute GRS is not fully understood. However, self-renewing cells at the base intestinal crypts play a crucial role, including intestine stem cells (ISCs) and daughter cells of the first few generations (Meena et al., 2022). Under physiological circumstances, ISCs self-renew, proliferate, and differentiate and thus maintain the intestinal epithelium integrity. After irradiation, these cells arrest the cell cycle and induce apoptosis (Li et al., 2021). When the number of ISCs and the production of daughter cells decrease substantially, the mucosal barrier that separates the intestinal content from the gastrointestinal tissue breaks down. This results in severe diarrhea, dehydration, electrolyte imbalance, and translocation of gastrointestinal pathogens and toxins into the body (Lu et al., 2019).

Acute GRS management is primarily symptomatic. It usually combines antiemetics, antidiarrheal drugs, rehydration, and antimicrobial prophylaxis and therapy (Lu et al., 2019). The fully developed syndrome has a poor prognosis. On the other hand, preclinical studies utilizing clinically available mitigators showed promising results. These mitigators also include different intestinotrophic substances, such as Teduglutide or Dinoprostone (Singh and Seed, 2020). Teduglutide is a dipeptidyl peptidase-resistant analog of glucagon-like peptide-2 (GLP-2). GLP-2 receptors have been localized to several intestinal cell types but not to the proliferating crypt cells. The GLP-2 actions have thus been associated with a complex network of indirect mediators activating diverse signaling pathways that enhance crypt cell proliferation and suppress apoptosis (Rowland and Brubaker, 2011). Gu et al. (2017) demonstrated that Teduglutide’s subcutaneous injection in specific pathogen-free Balb/c mice for 7 days prolonged survival of animals, decreased structural damage, down-regulated radiation-induced inflammatory responses, and promoted survival of crypt cells. Dinoprostone is prostaglandin E2 (PGE_2_), a lipid with pleiotropic effects. Both PGE_2_ and its long-acting analog 16,16-dimethyl PGE_2_ (dmPGE_2_) act via EP receptors (Okazaki et al., 2018). When administered before irradiation, both increase ISC survival and reduce crypt damage (Hanson and Thomas, 1983; Hanson and DeLaurentiis, 1987). PGE_2_ also promotes hematopoietic stem cell survival and hematopoietic recovery after radiation injury (Porter et al., 2013; Patterson et al., 2020). Patterson et al. (2020) defined a window of survival efficacy for single administration of dmPGE_2_ as within 3 h before and 6–30 h after total-body γ irradiation.

Insulin-like growth factor 1 (IGF-1) is another pleiotropic hormone. Its synthetic analog, mecasermin (brand name Increlex), is clinically used to treat growth failure in children (Bang et al., 2022). IGF-1 receptors are present in the intestine at different cell types, including ISCs (Van Landeghem et al., 2015). Systemically administered IGF-I increases crypt cell proliferation and expression of anti-apoptotic genes, particularly in the stem cell zone, which subsequently increases mucosal mass in mice (Dahly et al., 2002). After irradiation, IGF-1 signaling stimulates crypt regeneration (Bohin et al., 2020). IGF-1 also inhibits ionizing radiation (IR)-induced apoptosis of gastrointestinal vascular endothelial cells (Qiu et al., 2010). Their damage significantly affects the response of gastrointestinal tissues after irradiation (Lu et al., 2019). Howarth et al. (1997) implanted mini-osmotic pumps infusing IGF-1 into rats before whole-body irradiation by 10 Gy and observed accelerated intestinal mucosal recovery from radiation injury. However, the maximally tolerated dose for a single subcutaneous injection has not yet been published and tested for its radiation mitigation properties when administered in different dosage regimens.

## 2 Material and Methods

### 2.1 Animals

All experiments were performed with female C57Bl/6 mice (age 12–16 weeks, weight 18.8–23.2 g; Velaz, Unetice, Czech Republic). Mice were housed in an accredited facility (temperature 22 ± 2°C, 50 ± 10% relative humidity, with lights from 7:00 to 19:00 h; accreditation number č. j. 69233/2015-MZE-17214; Faculty of Military Health Sciences) and allowed access to tap water and standard food DOS-2B (BIOPO spol. s.r.o., Brno, Czech Republic) *ad libitum*. The animals were acclimatized for 14 days before starting the experiments. All experiments in this study were approved by the Ethics Committee (Faculty of Military Health Sciences, Hradec Kralove, Czech Republic) and were conducted following the Animal Protection Law and Animal Protection Regulations.

### 2.2 Safety of IGF-1

Animals were randomly divided into four groups (*n* = 6). Recombinant human IGF-1 (Increlex; Ipsen Pharma, Boulogne-Billancourt, France) was administered subcutaneously (s.c.) at a dose of 0.2, 1, or 2 mg/kg to animals fasting for 12 h. Physiological saline (B Braun Melsungen AG, Melsungen, Germany) was used to dilute the growth factor and as a negative control. Blood was collected using the tail incision method at 0 (immediately before), 0.5, 1, 2, and 4 h after physiological saline or IGF-1 administration. Glucose concentration in blood was measured using SD CodeFree blood glucose monitor and SD CodeFree Plus blood glucose test strips (both from SD Biosensor, Suwon, South Korea). During the experiment, the animals were observed for clinical signs of hypoglycemia.

Clinical signs and glycemic profiles were also monitored in a group of animals (*n* = 6) that were not fasting before but were restricted from feeding during the experiment (4 h). The animals were administered s.c. with IGF-1 at 1 mg/kg. Glycemia was measured at 0 (immediately before), 0.5, 1, 2, and 4 h after IGF-1 administration.

All irradiation experiments were performed in non-fasting animals with free access to food during the experiments.

### 2.3 Source of Ionizing Radiation

The source of gamma radiation was ^60^Co unit (Chirana, Prague, Czech Republic). The dosimetry was performed using an ionization chamber (Dosemeter PTW Unidos 1001, Serial No. 11057, with ionization chamber PTW TM 313, Serial No. 0012; RPD Inc., Albertville, MN, United States).

### 2.4 Irradiation Procedure

Before IR treatments, animals were anesthetized using a solution of Rometar (20 mg/ml; Bioveta, Ivanovice na Hane, Czech Republic), Narketan (50 mg/ml; Vetoquinol, Prague, Czech Republic), and physiological saline in the volume ratio 1:3:12. This solution was administered intramuscularly at a dose of 10 ml/kg. The anesthetized animals were placed into a Plexiglas box (VLA JEP, Hradec Kralove, Czech Republic) and irradiated by a single dose of IR delivered from back to front at a dose rate of 0.81 Gy/min (survival experiment and assessment of jejunal and lung damage) or 0.27 Gy/min (assessment of different IGF-1 dosage regimens) with a target distance of 1 m. In both experiments, the head and neck were shielded with 10 cm thick lead bricks.

### 2.5 Experimental Setup of Ionizing Radiation Experiments

In survival experiments, the mice were randomly divided into 6 groups (*n* = 20) and irradiated by 14, 14.5, or 15 Gy. IGF-1 was administered s.c. at 1 mg/kg 1, 24, and 48 h after irradiation. Physiological saline was used to dilute the growth factor and as a negative control. The survival of animals was monitored daily.

Simultaneously, we assessed the effect of IGF-1 on IR-induced jejunum and lung damage. For this, mice were randomly divided into 6 groups (*n* = 8), irradiated by 12, 14, or 15 Gy, and administered s.c. with IGF-1 (1 mg/kg) 1, 24, and 48 h after irradiation. Physiological saline was used to dilute IGF-1 and for the control group. Four hours before euthanasia, animals were intraperitoneally injected with 5-bromo-2′-deoxyuridine (BrdU, 100 mg/kg; Merck, Kenilworth, NJ, United States) diluted in physiological saline. After deep narcotization in the CO_2_ atmosphere at 84 h after irradiation, samples from the jejunum (5–6 cm from the pyloric ostium) and lung were collected and fixed with 10% neutral buffered formalin (Chemapol, Prague, Czech Republic).

Finally, we evaluated the effect of six different IGF-1 dosage regimens (à 1 mg/kg; single and multiple) on IR-induced gastrointestinal damage and peripheral blood changes. In this experiment, the animals were randomly divided into 16 groups (*n* = 6), irradiated by 12 or 14 Gy, and administered s.c. with IGF-1 according to the experimental setup presented in Table 1. Physiological saline was used to dilute IGF-1 and for the control group. 6 days after the irradiation, the animals were deeply narcotized in the CO_2_ atmosphere, and the thorax and abdominal cavity were opened. Venous blood was collected from the right heart ventricle into heparinized tubes (Scanlab Systems, Prague, Czech Republic). Samples from the duodenum, jejunum (0.5–1 cm, 5–6 cm from the pyloric ostium, respectively), and ileum (1–2 cm from the ileocecal valve) were collected and fixed with 10% neutral buffered formalin (Chemapol, Prague, Czech Republic).

**TABLE 1:** The experimental setup used to evaluate the different IGF-1 dosage regimens on the mitigation of ionizing radiation-induced gastrointestinal damage and peripheral blood changes.

Group	Dose (Gy)	IGF-1^a^	Group	Dose (Gy)	IGF-1^a^
1	0	-	9	0	-
2	12	-	10	14	-
3	12	1 (1 h)	11	14	1 (1 h)
4	12	2 (1, 24 h)	12	14	2 (1, 24 h)
5	12	3 (1, 24, 48 h)	13	14	3 (1, 24, 48 h)
6	12	4 (1, 24, 48, 72 h)	14	14	4 (1, 24, 48, 72 h)
7	12	5 (1, 24, 48, 72, 96 h)	15	14	5 (1, 24, 48, 72, 96 h)
8	12	3 (24, 48, 72 h)	16	14	3 (24, 48, 72 h)

a Number of doses (time of administration). IGF-1, was administered subcutaneously at a dose of 1 mg/kg.

### 2.6 Staining of Samples

#### 2.6.1 Hematoxylin-Eosin

According to the previously published procedure (Pejchal et al., 2012), formalin-fixed samples were processed and stained with hematoxylin and eosin (Merck).

#### 2.6.2 Detection of BrdU Positive Cells

Dewaxed 5 μm thick sections first underwent DNA hydrolysis in 2 M HCl (Merck) for 1 h at 37°C. Subsequently, the sections were neutralized in 0.1 M sodium borate buffer (pH 8.5; Merck) for 10 min at room temperature and washed three times in phosphate-buffered saline (PBS; Merck). BrdU incorporation was then detected using a standard peroxidase technique (Pejchal et al., 2012). In short, after blocking the endogenous peroxidase activity for 20 min, the tissue sections were incubated for 1 h with rat monoclonal anti-BrdU antibody (1 μg/ml; clone BU1/75 [ICR1], Abcam, Cambridge, United Kingdom). As a secondary antibody, pre-diluted ready-to-use goat anti-rat antibody-HPR polymer (ab214882; Abcam) was applied for 20 min. Finally, 0.05% 3,3′-diaminobenzidine tetrahydrochloride-chromogen solution (Merck) in PBS containing 0.02% hydrogen peroxide was added for 10 min to visualize the antigen-antibody complex.

#### 2.6.3 Detection of Chloroacetate Esterase-Positive Cells

Chloroacetate esterase is considered specific for cells of granulocytic lineage. To detect chloroacetate esterase-positive cells, dewaxed and hydrated 5 μm thick sections were stained using a naphthol AS-D chloroacetate esterase kit according to the manufacturer (Cat. No. 91C-1KT; Merck) instructions. The samples were mounted into an ImmunoHistoMount aqueous-based mounting medium (Merck).

### 2.7 Evaluation of the Jejunum and Lung Damage

#### 2.7.1 Jejunum

In the jejunum, we first performed a histopathological analysis in hematoxylin-eosin-stained samples. The samples were semiquantitatively scored for the loss of epithelial continuity, edema, and granulocyte infiltration (Table 2). Additionally, the number of villi per circumference, their length, number of surviving crypts per circumference, and the amount of chloroacetate esterase positive cells were scored.

**TABLE 2:** A semiquantitative score of histopathological changes in the intestine.

Parameter	Score			
	0	1	2	3
loss of epithelial continuity^a^	not present	<5 microerosions	≥5 microerosions	confluent changes
edema^b^	not present	mild	moderate	severe
granulocyte infiltration^c^	<3	≥3	≥10	≥50

a Evaluated in the whole cross-section.

b Number of neutrophile granulocytes per microscopic field at 400fold original magnification.

c Mild: subepithelial edema in <25% villi; moderate: subepithelial edema in ≥25% villi or cellular edema of <25% villous cells, or edema of <25% of lamina propria or submucosa; severe: cellular edema of ≥25% villous cells, or edema of ≥25% of lamina propria or submucosa.

A villus was judged as a villous-like structure containing at least 20 nucleated cells. The number of villi per circumference was counted in the whole cross-section of hematoxylin-eosin stained samples at ×200 magnification. Three cross-sections were evaluated for each animal, and their values were averaged.

The length of villi was assessed in the same samples using a BX-51 microscope (Olympus, Tokyo, Japan) and the ImagePro 5.1 computer image analysis system (Media Cybernetics, Bethesda, MD, United States). Lengths of 10 randomly selected villi were measured under ×160 magnification.

The amount of surviving crypts per circumference was counted in the whole cross-section of BrdU-stained samples at ×400 magnification. Only transversely sectioned crypts with ≥10 BrdU positive cells were considered as surviving. Three cross-sections were evaluated for each animal, and their values were averaged.

Chloroacetate esterase-positive cells were counted only in sub-villar mucose per microscopic field (centered in the middle of the field) to avoid the effect of different lengths of villi. Ten randomly selected microscopic fields were counted for each animal at ×400 magnification.

#### 2.7.2 Lung

Histopathological analysis was also done in the lung, scoring cellularity, inflammation, hyperemia, and edema (Table 3). Subsequently, the airness of the tissue and the number of chloroacetate esterase-positive cells were measured.

**TABLE 3:** A semiquantitative score of histopathological changes in the intestine.

Parameter	Score			
	0	1	2	3
cellularity	standard	mildly increase	moderately increased	severely increased
granulocyte infiltration^a^	0–10 per m.f	10–20 per m.f	≥20 per m.f	diffuse infiltration
edema^b^	not present	mild	moderate	severe
hyperemia	not present	mild	moderate	severe

m.f.–microscopic field.

a Number of neutrophile granulocytes per microscopic field at 400fold original magnification.

b Mild: mild intraseptal edema; moderate: moderate intraseptal edema with <10% alveoly with intraalveolar edema; severe: moderate intraseptal edema with ≥10% alveoly with intraalveolar edema.

The airness was assessed in hematoxylin-eosin-stained samples using a BX-51 microscope and the ImagePro 5.1 computer image analysis system. Ten microscopic fields at ×400 magnification were randomly selected from each animal. The airness of the tissue was expressed as the percentage of the bright area in the microscopic field defined in the red/green/blue scale: red 220–255, green 220–255, and blue 220–255, where 0 is black and 255 is white.

Finally, the number of chloroacetate esterase-positive cells was counted in ten randomly selected microscopic fields at ×400 magnification for each mouse.

### 2.8 Evaluation of Different Regimens

#### 2.8.1 Blood

Collected venous blood was promptly analyzed using ABX Pentra 60C + hemoanalyzer (Horiba, Kyoto, Japan). All samples were measured three times, and their values were averaged.

#### 2.8.2 Intestine

In hematoxylin-eosin-stained intestinal samples, the histopathological analysis, the number of villi per circumference, their length (all three parameters were measured similarly to the previous model), and the amount of regenerating crypts per circumference, and their length were measured.

Regenerating crypts were defined as basophilic cell clusters of ≥10 epithelial cells (excluding Paneth cells), each with a prominent nucleus and little cytoplasm, lying close together and appearing crowded (Bhat et al., 2019). The number of regenerating crypts per circumference was counted in the whole cross-section at ×400 magnification. Only transversely sectioned crypts with ≥10 epithelial cells were considered as regenerating. Three cross-sections were evaluated for each animal, and their values were averaged.

The length of crypts was assessed by BX-51 microscope and the ImagePro 5.1 computer image analysis system. Lengths of 10 randomly selected crypts were measured under ×160 magnification.

### 2.9 Statistical Analysis

The Kaplan-Meier Survival Analysis with post hoc Log Rank test and Mann-Whitney test by SPSS statistics version 24 (IBM, Armonk, NY, United States) were used for the statistical analysis. Graphs were produced using GraphPad Prism software (version5.04, GraphPad Software Inc., San Diego, CA). The differences were considered significant when *p* ≤ 0.05.

## 3 Results

### 3.1 IGF-1 Safety Assessment

**FIGURE 1:**
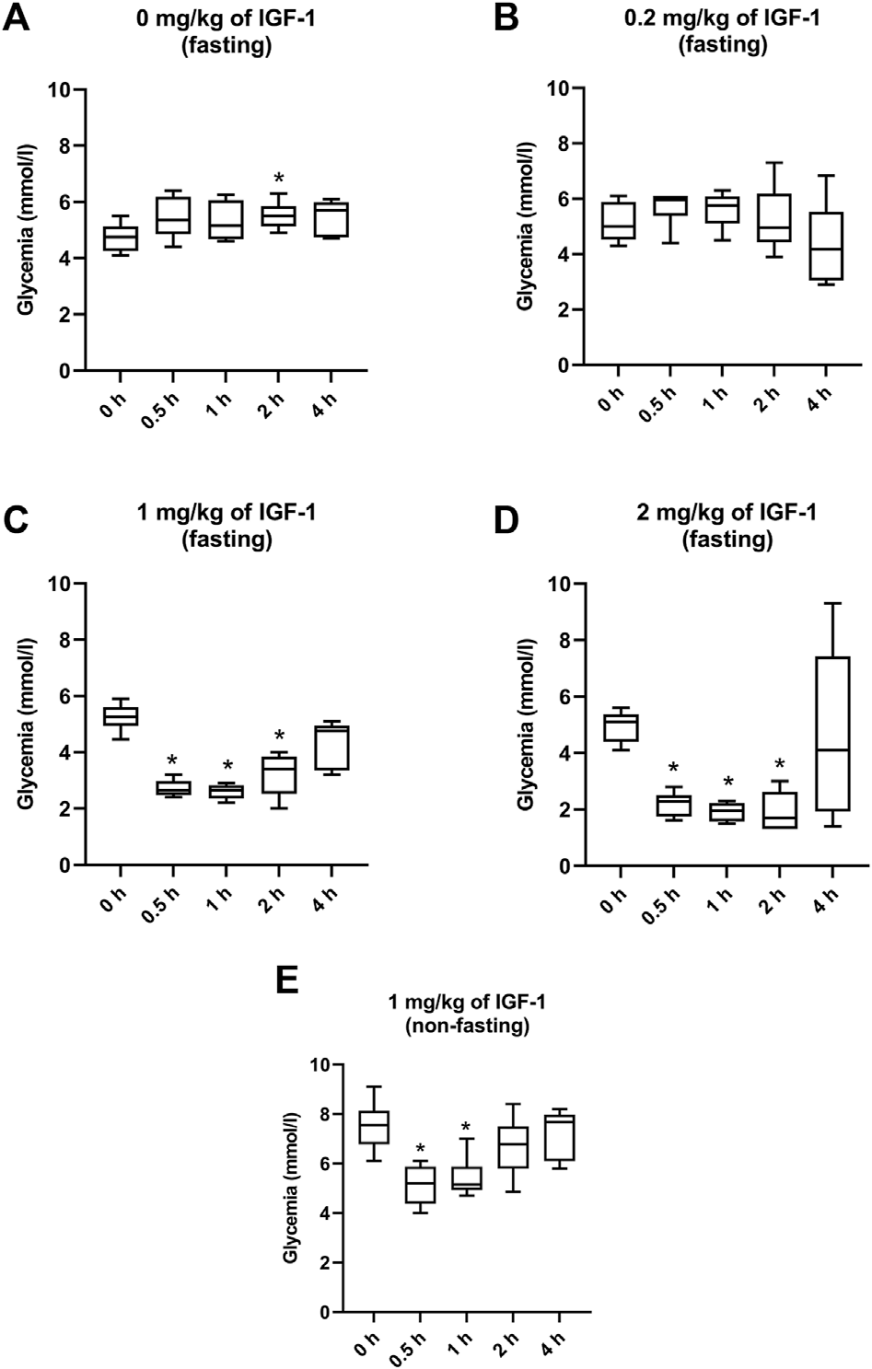
Effect of IGF-1 on mouse glycemia. **(A)** mice fasting for 12 h before the experiment administered with physiological saline. **(B)** mice fasting for 12 h before the experiment administered with IGF-1 at a dose of 0.2 mg/kg. **(C)** mice fasting for 12 h before the experiment administered with IGF-1 at a dose of 1 mg/kg. **(D)** mice fasting for 12 h before the experiment administered with IGF-1 at a dose of 2 mg/kg. **(E)** non-fasting mice administered with IGF-1 at a dose of 1 mg/kg.

In the control of fasting animals (0 mg/kg), the experimental procedure (handling and blood collection) significantly increased blood glucose levels by 16% in the 2-h interval (Figure 1A). The 0.2 mg/kg dose did not affect glycemia nor induced any clinical alteration (Figure 1B). After administration of IGF-1, the dose of 1 mg/kg significantly decreased median glycemia by 50, 50, and 36% at 0.5, 1, and 2 h, respectively (Figure 1C). Although acute hypoglycemia (<3 mmol/L) was recorded in 5 of 6 animals, no clinical symptoms associated with hypoglycemia were observed. The dose of 2 mg/kg decreased median glycemia by 55, 62, and 67% at 0.5, 1, and 2 h, respectively (Figure 1D). Mice displayed spatial segregation, hypoactivity, and decreased reactivity to external stimulation. Two mice experienced seizures. The maximum tolerated dose (MTD) was therefore established at 1 mg/kg.

In animals that were not fasting before but fasting during the experiment, 1 mg/kg of IGF-1 decreased median blood glucose levels by 31 and 32% at 0.5 and 1 h after the administration, respectively (Figure 1E). No clinical alterations were observed in this group.

### 3.2 Effect of IGF-1 on Animal Survival After Irradiation by 14, 14.5, and 15 Gy With Head and Neck Shielded

**FIGURE 2:**
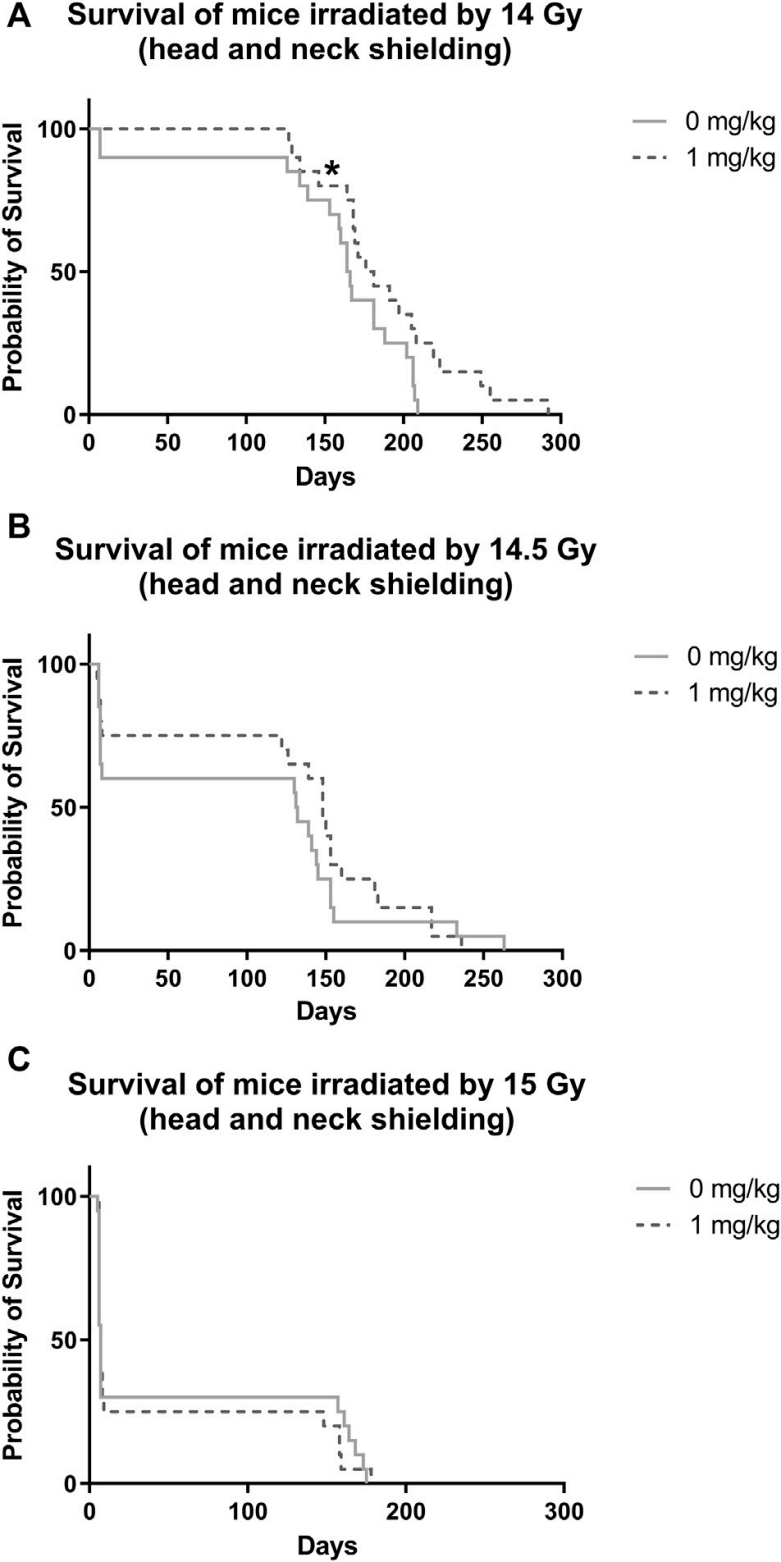
Effect of IGF-1 at 1 mg/kg on irradiated mice’s survival with shielded head and neck. **(A)** 14 Gy. **(B)** 14.5 Gy. **(C)** 15 Gy. Significantly different when compared with the solely irradiated group receiving no treatment: *p ≤ 0.05.

After irradiation by 14 Gy, survival significantly increased in animals administered with IGF-1 (median 176 days, 95% confidence interval [CI] = 154–198 days) when compared with control receiving only physiological saline (median = 164 days, 95% CI = 155–173 days; Figure 2A, Supplementary Table S1). We did not observe any significant differences between irradiated control receiving no treatment and IGF-1 administered groups after irradiation by 14.5 and 15 Gy (Figures 2B,C).

### 3.3 Effect of IGF-1 (MTD) on Jejunal and Lung Damage in Mice 84 h After Irradiation by 12, 14, and 15 Gy With Head and Neck Shielded

**FIGURE 3:**
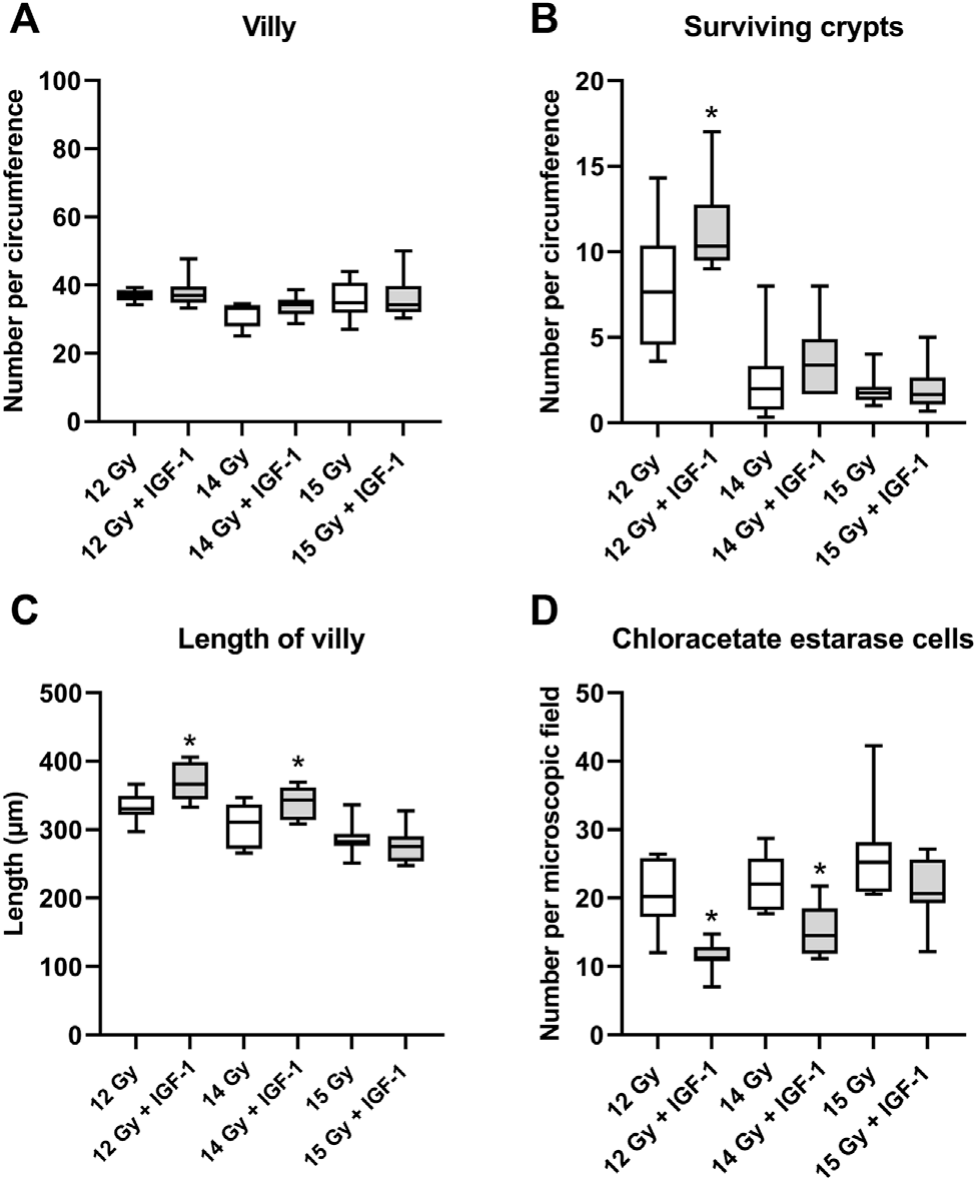
Effect of IGF-1 at 1 mg/kg in the jejunum of mice irradiated by 12, 14, and 15 Gy with shielded head and neck. **(A)** number of villy. **(B)** number of surviving crypts. **(C)** length of villy. **(D)** chloroacetate esterase-positive cells in the subvillar mucose per centered microscopic field at ×400 magnification. Significantly different when compared with the solely irradiated group receiving no treatment: *p ≤ 0.05.

In the jejunum, IGF-1 treatment increased the length of villi and the number of surviving crypts while reducing the amount of chloroacetate esterase positive cells in the tissue at 12 Gy (by 10, 35, and 45%, respectively; Supplementary Figure S1). After irradiation by 14 Gy, the therapy only prolonged the villi and decreased the number of infiltrating chloroacetate esterase positive cells (by 10 and 34%, respectively; Figure 3). IGF-1 did not affect any histopathological parameter (Supplementary Figure S2) or the number of villi per circumference (Figure 3A).

**FIGURE 4:**
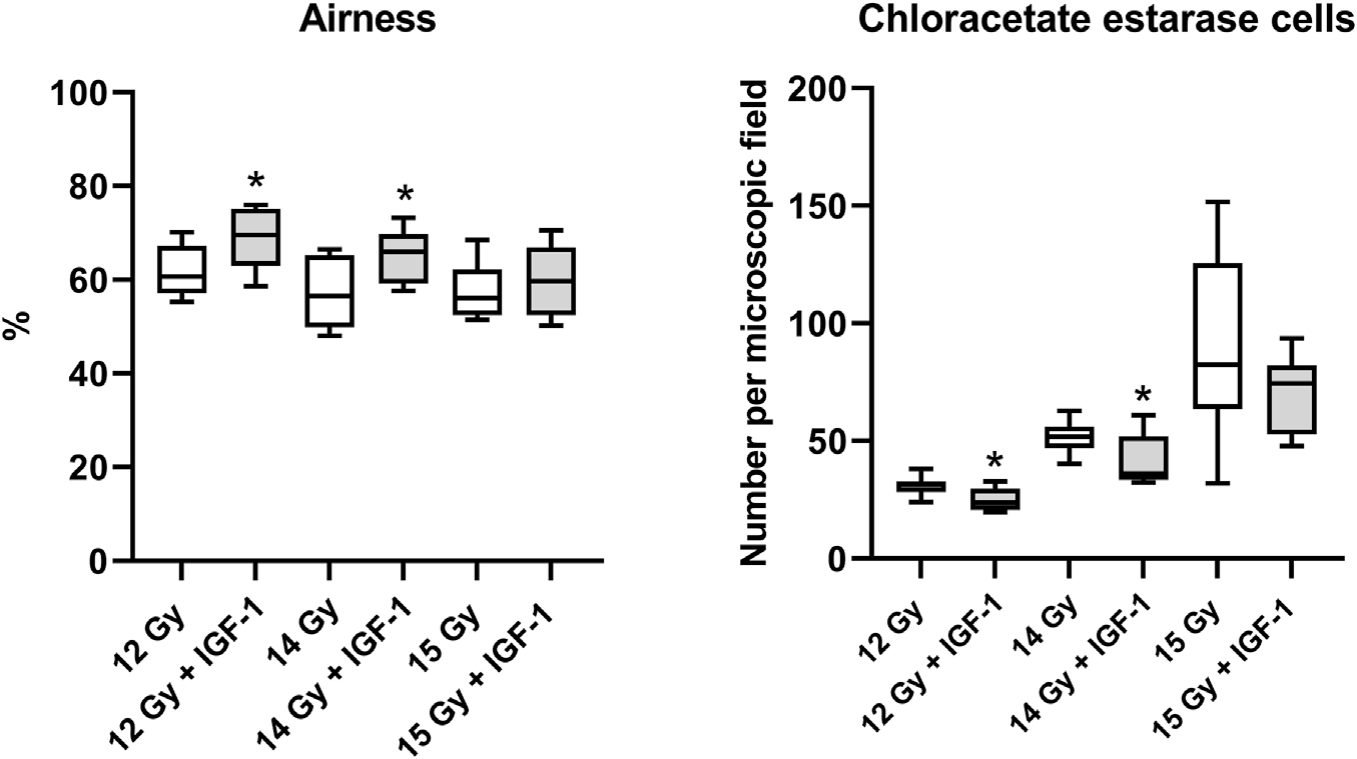
Effect of IGF-1 at 1 mg/kg in the lung of mice irradiated by 12, 14, and 15 Gy with shielded head and neck. A: airness of the tissue. B: chloroacetate esterase-positive cells per microscopic field at ×400 magnification. Significantly different when compared with the solely irradiated group receiving no treatment: *p ≤ 0.05.

In the lung, IGF-1 did not significantly affect any histopathological parameter (Supplementary Figure S3). Still, it increased airness while reducing the amount of chloroacetate esterase positive cells at 12 Gy (by 14 and 24%; Supplementary Figure S4) and 14 Gy (by 17 and 30%, respectively; Figure 4).

### 3.4 Effect of Different IGF-1 Therapeutical Regimens in Duodenum, Jejunum, and Ileum of Animals Irradiated Six Days After Irradiation by 12 and 14 Gy With Head and Neck Shielded

#### 3.4.1 Histopathological Assessment

IR at 12 Gy did not induce any significant alterations. At 14 Gy, we observed edema in the duodenum, jejunum, and ileum and inflammation in the duodenum and jejunum. Compared with solely irradiated groups, IGF-1 therapy did not significantly affect the histopathological scores of IR-induced changes (Supplementary Figures S5–S7).

#### 3.4.2 Number of Villy

**FIGURE 5:**
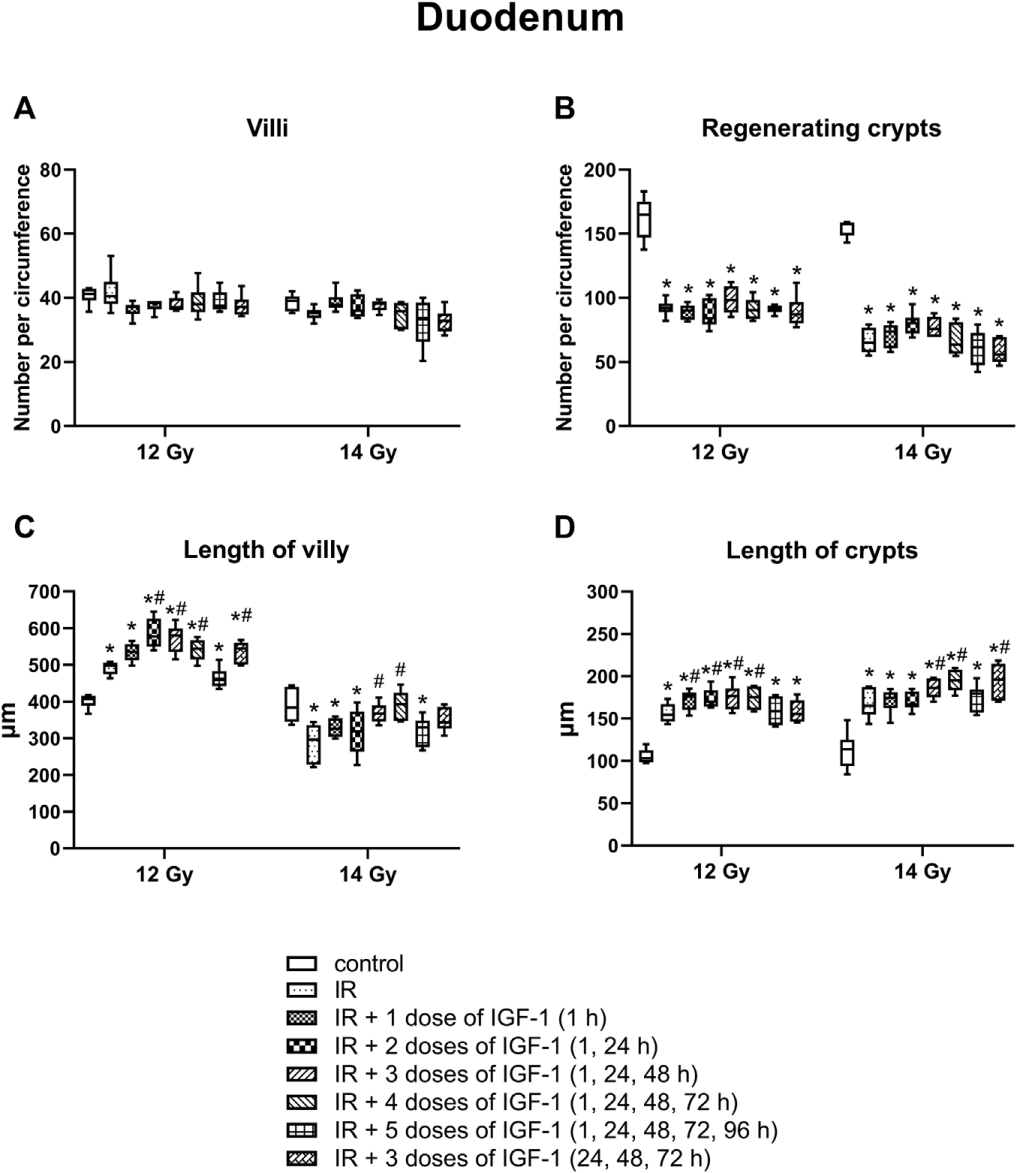
Effect of different IGF-1 (1 mg/kg) therapeutical regimens on the duodenum of mice irradiated by 12 or 14 Gy with shielded head and neck. **(A)** number of villy. **(B)** number of surviving crypts (microcolony assay). **(C)** length of villy. **(D)** length of crypts. Significantly different when compared with the non-irradiated control: *p ≤ 0.05. Significantly different when compared with the solely irradiated group receiving no treatment: ^#^ *p* ≤ 0.05.

**FIGURE 6:**
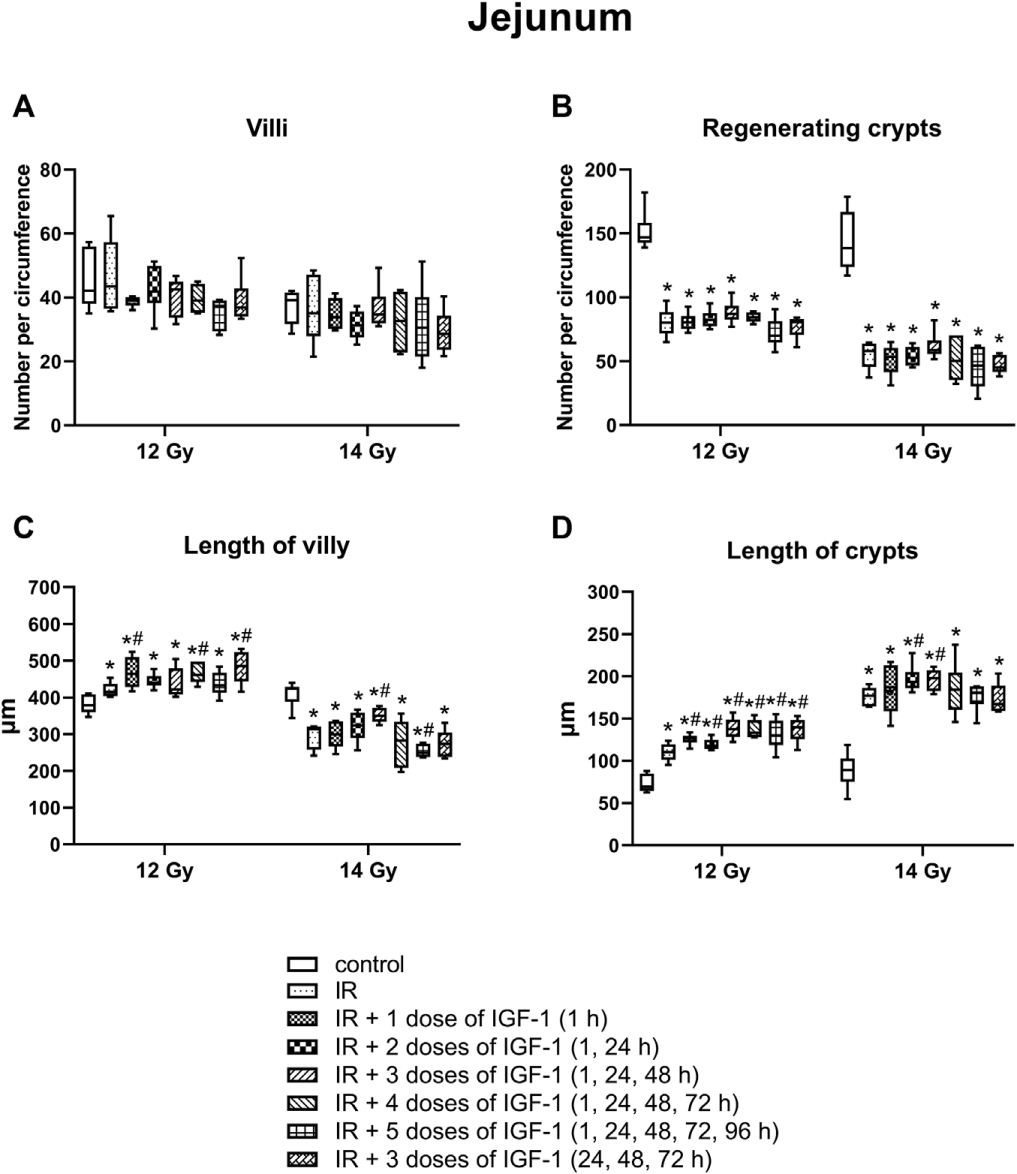
Effect of different IGF-1 (1 mg/kg) therapeutical regimens on jejunum of mice irradiated by 12 or 14 Gy with shielded head and neck. **(A)** number of villy. **(B)** number of surviving crypts (microcolony assay). **(C)** length of villy. **(D)** length of crypts. Significantly different when compared with the non-irradiated control: *p ≤ 0.05. Significantly different when compared with the solely irradiated group receiving no treatment: ^#^ *p* ≤ 0.05.

**FIGURE 7:**
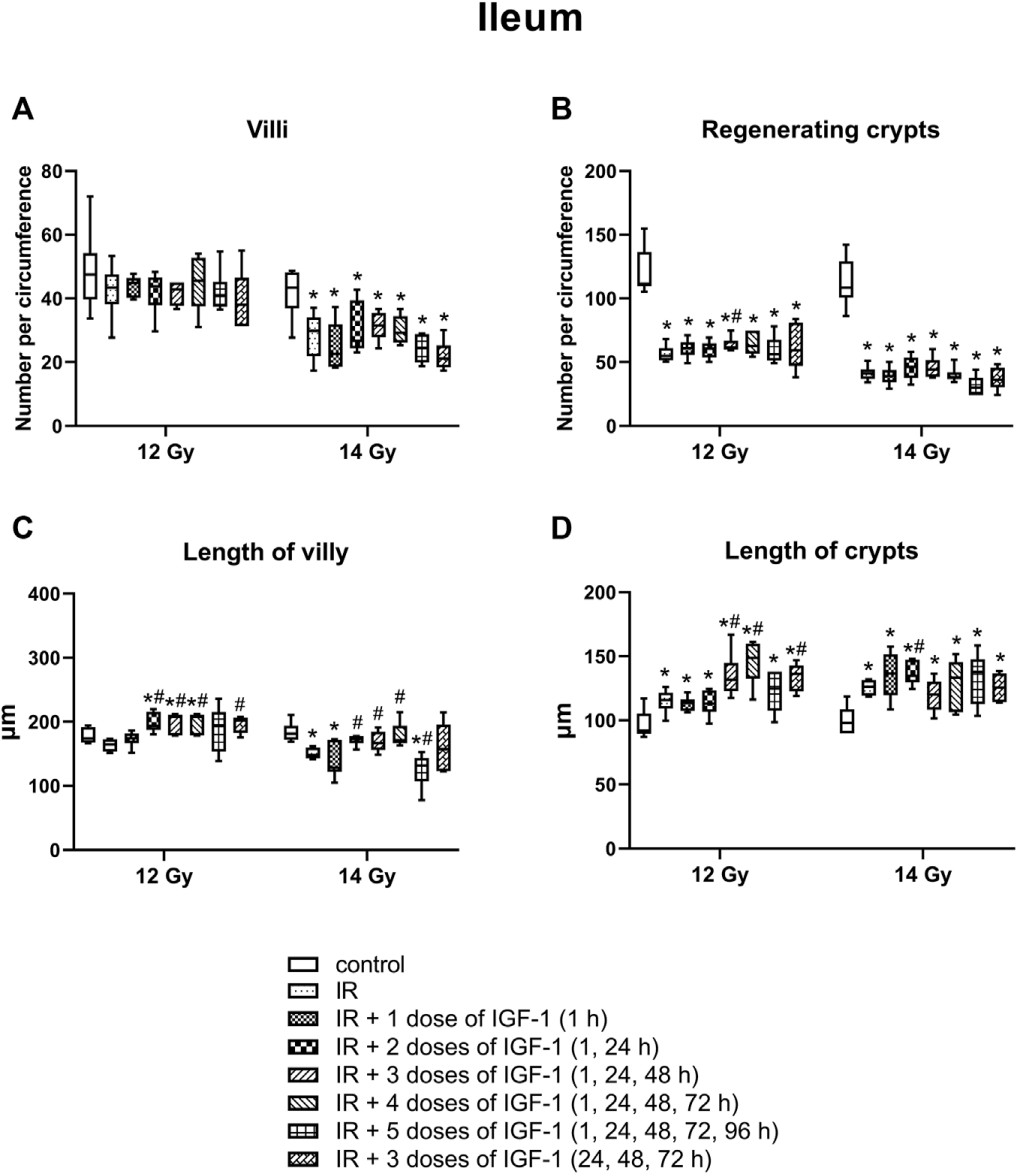
Effect of different therapeutical regimens of IGF-1 (1 mg/kg) on ileum of mice irradiated by 12 or 14 Gy with shielded head and neck. **(A)** number of villy. **(B)** number of surviving crypts (microcolony assay). **(C)** length of villy. **(D)** length of crypts. Significantly different when compared with the non-irradiated control: *p ≤ 0.05. Significantly different when compared with the solely irradiated group receiving no treatment: ^#^ *p* ≤ 0.05.

The median amount of villy significantly decreased only in the ileum by 31% of mice irradiated by 14 Gy. Different IGF-1 regimens did not show any therapeutical effect (Figures 5A–7A).

#### 3.4.3 Number of Regenerating Crypts

The median number of surviving crypts dropped by 44 and 59% in the duodenum (Figure 5B), by 51 and 62% in the jejunum (Figure 6B), and by 51 and 62% in the ileum (Figure 7B) after irradiation by 12 and 14 Gy, respectively. The therapeutical effect was noted only in the ileum of mice irradiated by 12 Gy. Administration of IGF-1 in 3 doses significantly increased the median value by 12%.

#### 3.4.4 Length of Villy

In the duodenum (Figure 5C), the median length of villy increased by 22% after irradiation by 12 Gy. Administration of 2, 3, and 4 doses of IGF-1 and 3 doses with later onset of administration prolonged median villy values by 16, 17, 9, and 9%, respectively. At 14 Gy, the length of villy significantly decreased by 23% in solely irradiated animals. Compared with this group, administration of 3 and 4 doses of IGF-1 prolonged villy by 24 and 32%, respectively.

In jejunum (Figure 6C), the parameter increased by 10% after irradiation by 12 Gy. The administration of 1, 4 doses of IGF-1 and 3 doses with later onset of administration further prolonged villy by 12, 11, and 17%, respectively. Irradiation by 14 Gy decreased the median length of villy in the jejunum by 26%. Compared with this group, administration of 3 doses of IGF-1 significantly prolonged villy by 12%, whereas their size decreased in the group administered with 5 doses by 21%.

In the ileum after 12 Gy irradiation (Figure 7C), the administration of 2, 3, and 4 doses of IGF-1 and 3 doses with later onset of administration prolonged median values by 18, 26, 45, 23%, respectively. At 14 Gy, the parameter decreased by 18% in solely irradiated animals. Compared with this group, administration of 2, 3, and 4 doses of IGF-1 prolonged villy by 17, 13, and 16%, respectively. By contrast, 5 doses of IGF-1 further decreased their size by 21%.

#### 3.4.5 The Length of Crypts

In the duodenum (Figure 5D), the crypts’ median length increased by 50% after 12 Gy. Compared with the solely irradiated group, administration of 1, 2, 3, and 4 doses of IGF-1 further prolonged crypts by 10, 10, 14, and 13%, respectively. At 14 Gy, the length of crypts significantly increased by 45% in solely irradiated animals. In comparison with this group, administration of 3 and 4 doses of IGF-1 and 3 doses with later onset of administration prolonged crypts by 13, 18, and 19%, respectively.

In jejunum (Figure 6D), the median of this parameter increased by 59% at 12 Gy. The administration of 1, 3, 4, and 5 doses of IGF-1 and 3 doses with later onset of administration further prolonged crypts by 15, 25, 21, 21, and 27%, respectively. Irradiation by 14 Gy increased jejunal crypts’ length by 99%. Compared with this group, administration of 2 and 3 doses of IGF-1 significantly prolonged crypts by 10 and 12%, respectively.

In the ileum (Figure 7D), the parameter increased by 26% after irradiation by 12 Gy. The administration of 3 and 4 doses of IGF-1 and 3 doses with later onset of administration prolonged crypts by 14, 28, and 17%, respectively. At 14 Gy, the parameter increased by 29% in solely irradiated animals. Compared with this group, administration of 2 doses of IGF-1 prolonged crypts by 10%.

### 3.5 Effect of Different IGF-1 Therapeutical Regimens on Blood Parameters in Animals Irradiated by 12 and 14 Gy With Head and Neck Shielded Evaluated Six Days After Irradiation

**FIGURE 8:**
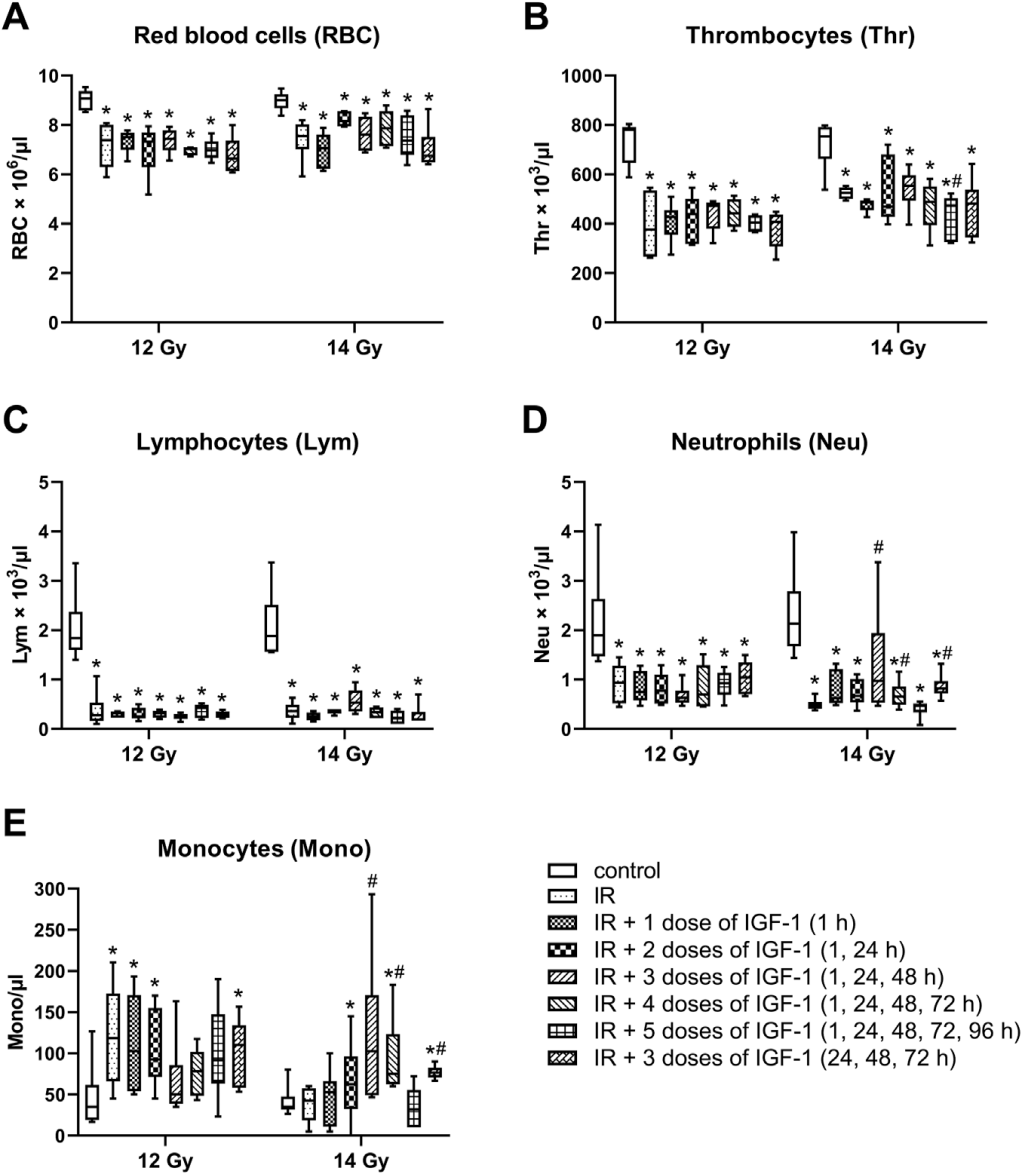
Effect of different IGF-1 (1 mg/kg) therapeutical regimens on blood hematological parameters of mice irradiated by 12 or 14 Gy with shielded head and neck. **(A)** red blood cells. **(B)** thrombocytes. **(C)** lymphocytes. **(D)** neutrophils. **(E)** monocytes. Significantly different when compared with the non-irradiated control: *p ≤ 0.05. Significantly different when compared with the solely irradiated group receiving no treatment: ^#^ *p* ≤ 0.05.

After irradiation by 12 and 14 Gy, median erythrocyte values significantly decreased by 19 and 16% (Figure 8A), thrombocytes by 52 and 30% (Figure 8B), lymphocytes by 85 and 81% (Figure 8C), and neutrophils by 50 and 78% (Figure 8D), respectively. By contrast, the monocyte median increased 3.4 fold in mice irradiated by 12 Gy (Figure 8E).

Compared with solely irradiated groups, IGF-1 did not affect blood parameters in animals irradiated by 12 Gy. At 14 Gy, the therapy with 3 and 4 doses of IGF-1 and 3 doses with later onset of administration increased median neutrophil values 2.0-, 1.4-, and 1.7 fold and median monocyte values 2.4-, 1.7-, and 1.6 fold, respectively. Five doses decreased the thrombocyte median by 10%.

## 4 Discussion

Administration of IGF-1 may induce severe hypoglycemia due to its molecular structure and functional similarity with insulin (Bang et al., 2022). This response seems dose-dependent (Woodall et al., 1991). Therefore, our study evaluated glycemic response to different single subcutaneous doses of IGF-1 in fasting female C57Bl/6J mice. The 1 mg/kg dose induced mild hypoglycemia but was clinically well tolerated. The brain is one of the first organs affected by hypoglycemia. Shortage of glucose in the brain cause prolonged reaction time, seizures, loss of consciousness, or death as the hypoglycemia progresses (Blaabjerg and Juhl, 2016). We did not observe any of these signs in this group. The 1 mg/kg dose seems even completely safe in non-fasting animals, possibly allowing further dose increase. However, due to gastrointestinal damage, diarrhea, and weight loss that develop early after high-dose irradiation, 1 mg/kg was established as the MTD under the current experimental settings.

In the next experiment, IGF-1 was tested to mitigate the lethality of mice exposed to high doses of IR with a shielded head and neck. The model spares sufficient bone marrow in the head and neck regions to prevent lethality from acute hematopoietic radiation syndrome. But still, it does not protect other regions against exposure. Besides the gastrointestinal tract, the lungs are particularly susceptible to high-dose irradiation. In female C57BL/6J mice, the lower threshold for developing lethal radiation lung injury after chest irradiation is 12 Gy (Jackson et al., 2016). Two clinical-pathological units may develop after exceeding this threshold. Acute radiation pneumonitis manifests in a dose-dependent manner 1–6 months after irradiation, while radiation fibrosis develops after 6 months. Although the two clinical-pathological units have different pathogenesis, both are associated with alveolar epithelial cell depletion (Lierova et al., 2018). By contrast, IGF-1 can stimulate the proliferation of alveolar epithelial cells type II and their differentiation into alveolar epithelial cells type I and modulates the inflammatory response of lung tissue (Zhang et al., 2022). Our study first investigated the potential of IGF-1 to affect IR-induced lethality. Since IGF-1 stimulates proliferation or cell viability in a dose-dependent manner (Yang et al., 2020; Hossain et al., 2021), the factor was administered at MTD. The initial regimen consisting of three doses followed cytokine guidelines established for other growth factors moderating epithelial tissue damage, such as KGF or EGF (Drouet and Hérodin, 2010; Pejchal et al., 2015). The results show that the therapy mitigated lethality induced by 14 Gy but could not protect against higher doses of IR. These findings corresponded with the second irradiation model. In this experiment, IGF-1 mitigated IR-induced morphological damage and inflammation in jejunum and lung after irradiation by 12 and 14 Gy but was ineffective at 15 Gy.

The final model aimed to achieve an optimal dosing regimen of the growth factor to mitigate IR-induced gastrointestinal damage. Tissue samples in this model were collected 6 days post-irradiation when signs of atrophy, inflammation, and regeneration can be observed in the gastrointestinal tract. Six different dosing regimens were tested. Five of them started at 1 h after irradiation. Potten and Grant (1998) recorded the intestine’s maximum apoptotic activity during 3–6 h after radiation by 1 Gy. Thus, it is necessary to start the application as soon as possible after irradiation to save as many stem cells and early progenitors as possible. IGF-1 generally supported the mucosal renewal. The elongation of crypts and villi indicates that the growth factor administration stimulated cellular proliferation in crypts and the production of new cells into the superficial compartment. However, individual dosing regimens showed different effects, with three- and four-dose regimens being the most effective (Table 4). The three-dose regimens even stimulated intestinal regeneration when the administration began 24 h after irradiation, but its potency decreased. By contrast, the efficacy of the five-dose regimen significantly declined. The mechanism explaining this finding remains unknown. The theoretical explanation might lay in the selective down-regulation of IGF-1 receptors. Long-term exposure to high doses of the growth factor may reduce signaling associated with proliferation, anti-apoptotic, and anti-inflammatory action and ultimately shift the environment towards pro-inflammatory status (Kenchegowda et al., 2018).

**TABLE 4:** An overview of IGF-1-induced significant changes in gastrointestinal tract irradiated by 12 and 14 Gy.

Parameter		12 Gy						14 Gy					
		1 D	2 D	3 D	4 D	5 D	3 D_L_	1 D	2 D	3 D	4 D	5 D	3 D_L_
Villi	duodenum		+	+	+		+			+	+		
	jejunum	+			+		+			+		-	
	ileum		+	+	+		+		+	+	+	-	
Crypts	duodenum	+	+	+	+					+	+		+
	jejunum	+		+	+		+		+	+			
	ileum			+^a^	+	+	+		+				
Total		4	4	5	6	3	5	0	3	5	3	-2	1
%^b^		6	7	16	21	4	16	0	6	12	11	-7	3

+ and - represent significant changes (increase and decrease, respectively) compared with solely irradiated groups. D: dosage regimens starting at 1 h after irradiation. D_L_: three-dose regimen beginning 24 h after irradiation.

^a^To sum percentage changes in the 3 D group irradiated by 12 Gy, the length of crypts in the ileum was increased by 12% due to a significantly higher number of surviving crypts.

b Average change per compartment.

Another goal of this experimental model was to assess the effect of IGF-1 on IR-induced hematological damage. Although the head and neck were shielded with lead, a significant portion of the bone marrow was exposed to ionizing radiation, reducing counts for all three types of blood cells. IGF-1, on the other hand, enhances the survival of bone marrow stem cells and stimulates the proliferation and differentiation of progenitor cells *in vitro* (Li et al., 1997; Ratajczak et al., 1998; Miyagawa et al., 2000; Aro et al., 2002). Chen et al. (2012) injected IGF-1 subcutaneously at a dose of 100 μg/kg twice daily for 7 days to BALB/c mice after whole-body irradiation by 5 Gy. They demonstrated that IGF-1 could promote overall hematopoietic recovery, having the earliest effect on leukocytes from the seventh day. Our results did not show any changes in hematological parameters in IGF-1-treated mice after irradiation by 12 Gy. Thus, the 6-days interval seems too soon to induce any effect. In this regard, the increase in monocytes and granulocytes observed in the three- and four-dose regimens or the loss of platelets found in the five-dose regimen are most possibly associated with gastrointestinal damage.

In conclusion, IGF-1 attenuates gastrointestinal damage, but the efficacy depends on several factors, including timing, dose, and dose regimen. The dose of 1 mg/kg administered daily in three to four consecutive days post-radiation exerted the highest potency in mice. Nonetheless, there may be limitations to our study. One lies in the fact that all experiments were conducted on female animals. This choice was based on a negligible risk of inter-female aggressivity in the C57Bl/6 strain compared to males (Parmigiani et al., 1999). Intra-group aggressivity could be crucial for high-dose irradiation models and (to some extent) concurrent immunosuppression. Although sex hormones affect IGF-1 signaling, testosterone seems to potentiate IGF-1 biological roles (Li and Li, 2014; Hughes et al., 2016), implying even higher effectivity in males. The second is associated with recommended doses of Increlex for humans, ranging from 0.04 to 0.12 mg/kg (Bang et al., 2022). Administration of higher doses is not entirely ruled out. But it would require specialized care with continuous glycemia monitoring and correction, most likely limiting the application in the field conditions. Further studies utilizing larger experimental animals seem necessary to optimize the dose and dosage regimen of IGF-1 to establish this growth factor as an effective countermeasure for large-scale radiation incidents.

## Data Availability

The original contributions presented in the study are included in the article/Supplementary Material, further inquiries can be directed to the corresponding author.

## References

[B1] Aro A. L. Savikko J. Pulkkinen V. von Willebrand E. (2002). Expression of Insulin-like Growth Factors IGF-I and IGF-II, and Their Receptors during the Growth and Megakaryocytic Differentiation of K562 Cells. Leuk. Res. 26 (9), 831–837. 10.1016/s0145-2126(02)00006-1 12127559

[B2] Bang P. Polak M. Perrot V. Sert C. Shaikh H. Woelfle J. (2022). Pubertal Timing and Growth Dynamics in Children With Severe Primary IGF-1 Deficiency: Results from the European Increlex® Growth Forum Database Registry. Front. Endocrinol. (Lausanne) 13, 812568. 10.3389/fendo.2022.812568 35250870 PMC8895479

[B3] Bhat K. Duhachek-Muggy S. Ramanathan R. Saki M. Alli C. Medina P. (2019). 1-[(4-Nitrophenyl)sulfonyl]-4-phenylpiperazine Increases the Number of Peyer’s Patch-Associated Regenerating Crypts in the Small Intestines after Radiation Injury. Radiotherapy Oncol. 132, 8–15. 10.1016/j.radonc.2018.11.011 PMC6400303 30825974

[B4] Blaabjerg L. Juhl C. B. (2016). Hypoglycemia-Induced Changes in the Electroencephalogram: An Overview. J. Diabetes Sci. Technol. 10 (6), 1259–1267. 10.1177/1932296816659744 27464753 PMC5094337

[B5] Bohin N. McGowan K. P. Keeley T. M. Carlson E. A. Yan K. S. Samuelson L. C. (2020). Insulin-like Growth Factor-1 and mTORC1 Signaling Promote the Intestinal Regenerative Response After Irradiation Injury. Cell. Mol. Gastroenterology Hepatology 10 (4), 797–810. 10.1016/j.jcmgh.2020.05.013 PMC7502577 32502530

[B7] Chen S. Xu Y. Wang S. Shen M. Chen F. Chen M. (2012). Subcutaneous Administration of rhIGF-I Post Irradiation Exposure Enhances Hematopoietic Recovery and Survival in BALB/c Mice. J. Radiat. Res. 53 (4), 581–587. 10.1093/jrr/rrs029 22843623 PMC3393355

[B8] Dahly E. M. Guo Z. Ney D. M. (2002). Alterations in Enterocyte Proliferation and Apoptosis Accompany TPN-Induced Mucosal Hypoplasia and IGF-I-Induced Hyperplasia in Rats. J. Nutr. 132 (7), 2010–2014. 10.1093/jn/132.7.2010 12097684

[B42] Drouet M. Hérodin F. (2010). Radiation Victim Management and the Haematologist in the Future: Time to Revisit Therapeutic Guidelines?. Int. J. Radiat. Biol. 86 (8), 636–648. 10.3109/09553001003789604 20597842

[B41] Gu J. Liu S. Mu N. Huang T. Zhang W. Zhao H. (2017). A DPP-IV-Resistant Glucagon-Like Peptide-2 Dimer With Enhanced Activity Against Radiation-Induced Intestinal Injury. J. Control. Release 260, 32–45. 10.1016/j.jconrel.2017.05.020 28522195

[B9] Hanson W. R. DeLaurentiis K. (1987). Comparison of *In Vivo* Murine Intestinal Radiation Protection by E-Prostaglandins. Prostaglandins 33 Suppl, 93–104. 10.1016/0090-6980(87)90052-9 3423276

[B10] Hanson W. R. Thomas C. (1983). 16, 16-dimethyl Prostaglandin E2 Increases Survival of Murine Intestinal Stem Cells when Given before Photon Radiation. Radiat. Res. 96 (2), 393–398. 10.2307/3576222 6647767

[B11] Hossain M. A. Adithan A. Alam M. J. Kopalli S. R. Kim B. Kang C. W. (2021). IGF-1 Facilitates Cartilage Reconstruction by Regulating PI3K/AKT, MAPK, and NF-kB Signaling in Rabbit Osteoarthritis. J. Inflamm. Res. 14, 3555–3568. 10.2147/JIR.S316756 34335042 PMC8318731

[B12] Howarth G. S. Fraser R. Frisby C. L. Schirmer M. B. Yeoh E. K. (1997). Effects of Insulin-like Growth Factor-I Administration on Radiation Enteritis in Rats. Scand. J. Gastroenterol. 32 (11), 1118–1124. 10.3109/00365529709002990 9399392

[B13] Hughes D. C. Stewart C. E. Sculthorpe N. Dugdale H. F. Yousefian F. Lewis M. P. (2016). Testosterone Enables Growth and Hypertrophy in Fusion Impaired Myoblasts that Display Myotube Atrophy: Deciphering the Role of Androgen and IGF-I Receptors. Biogerontology 17 (3), 619–639. 10.1007/s10522-015-9621-9 26538344 PMC4889645

[B14] Jackson I. L. Zhang Y. Bentzen S. M. Hu J. Zhang A. Vujaskovic Z. (2016). Pathophysiological Mechanisms Underlying Phenotypic Differences in Pulmonary Radioresponse. Sci. Rep. 6, 36579. 10.1038/srep36579 27845360 PMC5109047

[B16] Kenchegowda D. Legesse B. Hritzo B. Olsen C. Aghdam S. Kaur A. (2018). Selective Insulin-like Growth Factor Resistance Associated with Heart Hemorrhages and Poor Prognosis in a Novel Preclinical Model of the Hematopoietic Acute Radiation Syndrome. Radiat. Res. 190 (2), 164–175. 10.1667/RR14993.1 29809108 PMC6118398

[B17] Li W. Lin Y. Luo Y. Wang Y. Lu Y. Li Y. (2021). Vitamin D Receptor Protects against Radiation-Induced Intestinal Injury in Mice via Inhibition of Intestinal Crypt Stem/Progenitor Cell Apoptosis. Nutrients 13 (9), 2910. 10.3390/nu13092910 34578802 PMC8466099

[B18] Li Y. Li K. (2014). Osteocalcin Induces Growth Hormone/insulin-like Growth Factor-1 System by Promoting Testosterone Synthesis in Male Mice. Horm. Metab. Res. 46 (11), 768–773. 10.1055/s-0034-1371869 24691732

[B19] Li Y. M. Schacher D. H. Liu Q. Arkins S. Rebeiz N. McCusker R. H. (1997). Regulation of Myeloid Growth and Differentiation by the Insulin-like Growth Factor I Receptor. Endocrinology 138 (1), 362–368. 10.1210/endo.138.1.4847 8977425

[B20] Lierova A. Jelicova M. Nemcova M. Proksova M. Pejchal J. Zarybnicka L. (2018). Cytokines and Radiation-Induced Pulmonary Injuries. J. Radiat. Res. 59 (6), 709–753. 10.1093/jrr/rry067 30169853 PMC6251431

[B21] Lu L. Li W. Chen L. Su Q. Wang Y. Guo Z. (2019). Radiation-induced Intestinal Damage: Latest Molecular and Clinical Developments. Future Oncol. 15 (35), 4105–4118. 10.2217/fon-2019-0416 31746639

[B22] Meena S. K. Joriya P. R. Yadav S. M. Kumar R. Meena P. Patel D. D. (2022). Modulation of Radiation-Induced Intestinal Injury by Radioprotective Agents: a Cellular and Molecular Perspectives. Rev. Environ. Health. In press. 10.1515/reveh-2021-0108 35438851

[B23] Miyagawa S. Kobayashi M. Konishi N. Sato T. Ueda K. (2000). Insulin and Insulin-like Growth Factor I Support the Proliferation of Erythroid Progenitor Cells in Bone Marrow through the Sharing of Receptors. Br. J. Haematol. 109 (3), 555–562. 10.1046/j.1365-2141.2000.02047.x 10886204

[B24] Parmigiani S. Palanza P. Rogers J. Ferrari P. F. (1999). Selection, Evolution of Behavior and Animal Models in Behavioral Neuroscience. Neurosci. Biobehav. Rev. 23 (7), 957–969. 10.1016/s0149-7634(99)00029-9 10580310

[B25] Patterson A. M. Liu L. Sampson C. H. Plett P. A. Li H. Singh P. (2020). A Single Radioprotective Dose of Prostaglandin E2 Blocks Irradiation-Induced Apoptotic Signaling and Early Cycling of Hematopoietic Stem Cells. Stem Cell. Rep. 15 (2), 358–373. 10.1016/j.stemcr.2020.07.004 PMC7419738 32735825

[B26] Pejchal J. Novotný J. Mařák V. Osterreicher J. Tichý A. Vávrová J. (2012). Activation of P38 MAPK and Expression of TGF-β1 in Rat Colon Enterocytes after Whole Body γ-irradiation. Int. J. Radiat. Biol. 88 (4), 348–358. 10.3109/09553002.2012.654044 22233094

[B27] Pejchal J. Šinkorová Z. Tichý A. Kmochová A. Ďurišová K. Kubelková K. (2015). Attenuation of Radiation-Induced Gastrointestinal Damage by Epidermal Growth Factor and Bone Marrow Transplantation in Mice. Int. J. Radiat. Biol. 91 (9), 703–714. 10.3109/09553002.2015.1054528 25994811

[B28] Porter R. L. Georger M. A. Bromberg O. McGrath K. E. Frisch B. J. Becker M. W. (2013). Prostaglandin E2 Increases Hematopoietic Stem Cell Survival and Accelerates Hematopoietic Recovery after Radiation Injury. Stem Cells 31 (2), 372–383. 10.1002/stem.1286 23169593 PMC3580384

[B29] Potten C. S. Grant H. K. (1998). The Relationship between Ionizing Radiation-Induced Apoptosis and Stem Cells in the Small and Large Intestine. Br. J. Cancer 78 (8), 993–1003. 10.1038/bjc.1998.618 9792141 PMC2063142

[B30] Qiu W. Leibowitz B. Zhang L. Yu J. (2010). Growth Factors Protect Intestinal Stem Cells from Radiation-Induced Apoptosis by Suppressing PUMA through the PI3K/AKT/p53 axis. Oncogene 29 (11), 1622–1632. 10.1038/onc.2009.451 19966853 PMC3076086

[B31] Ratajczak J. Zhang Q. Pertusini E. Wojczyk B. S. Wasik M. A. Ratajczak M. Z. (1998). The Role of Insulin (INS) and Insulin-like Growth Factor-I (IGF-I) in Regulating Human Erythropoiesis. Studies *In Vitro* under Serum-free Conditions-Ccomparison to Other Cytokines and Growth Factors. Leukemia 12 (3), 371–381. 10.1038/sj.leu.2400927 9529132

[B32] Rowland K. J. Brubaker P. L. (2011). The "cryptic" Mechanism of Action of Glucagon-like Peptide-2. Am J Physiol Gastrointest Liver PhysiolGastrointestinal Liver Physiology 301 (1), G1–G8. 10.1152/ajpgi.00039.2011 21527727

[B33] Sakai T. Hara J. Yamamura K. Okazaki A. Ohkura N. Sone T. (2018). Role of Prostaglandin I2 in the Bronchoconstriction-Triggered Cough Response in guinea Pigs. Exp. Lung Res. 44, 455–463. 10.1016/j.pupt.2017.09.00310.1080/01902148.2019.1590883 30931647

[B34] Singh V. K. Seed T. M. (2020). Pharmacological Management of Ionizing Radiation Injuries: Current and Prospective Agents and Targeted Organ Systems. Expert Opin. Pharmacother. 21 (3), 317–337. 10.1080/14656566.2019.1702968 31928256 PMC6982586

[B35] Van Landeghem L. Santoro M. A. Mah A. T. Krebs A. E. Dehmer J. J. McNaughton K. K. (2015). IGF1 Stimulates Crypt Expansion via Differential Activation of 2 Intestinal Stem Cell Populations. FASEB J. 29 (7), 2828–2842. 10.1096/fj.14-264010 25837582 PMC4478798

[B38] Woodall S. M. Breier B. H. O'Sullivan U. Gluckman P. D. (1991). The Effect of the Frequency of Subcutaneous Insulin-like Growth Factor-1 Administration on Weight Gain in Growth Hormone Deficient Mice. Horm. Metab. Res. 23 (12), 581–584. 10.1055/s-2007-1003760 1778592

[B39] Yang L. Tan Z. Li Y. Zhang X. Wu Y. Xu B. (2020). Insulin-like Growth Factor 1 Promotes Proliferation and Invasion of Papillary Thyroid Cancer through the STAT3 Pathway. J. Clin. Laboratory Analysis 34 (12), e23531. 10.1002/jcla.23531 PMC7755808 32851683

[B40] Zhang S. Luan X. Li H. Jin Z. (2022). Insulin-like Growth Factor-1: A Potential Target for Bronchopulmonary Dysplasia Treatment (Review). Exp. Ther. Med. 23 (3), 191. 10.3892/etm.2022.11114 35126694 PMC8794548

