# Supplementary material for: Mitigation of Ionizing Radiation-Induced Gastrointestinal Damage by Insulin-Like Growth Factor-1 in Mice
Source: Front Pharmacol. 2022 Jun 29;13:663855. doi: 10.3389/fphar.2022.663855 (PMC9277384; doi:10.3389/fphar.2022.663855)
Supplement: Supplementary file 1 [file DataSheet1.docx]

Supplementary Material

# Supplementary Figures

**
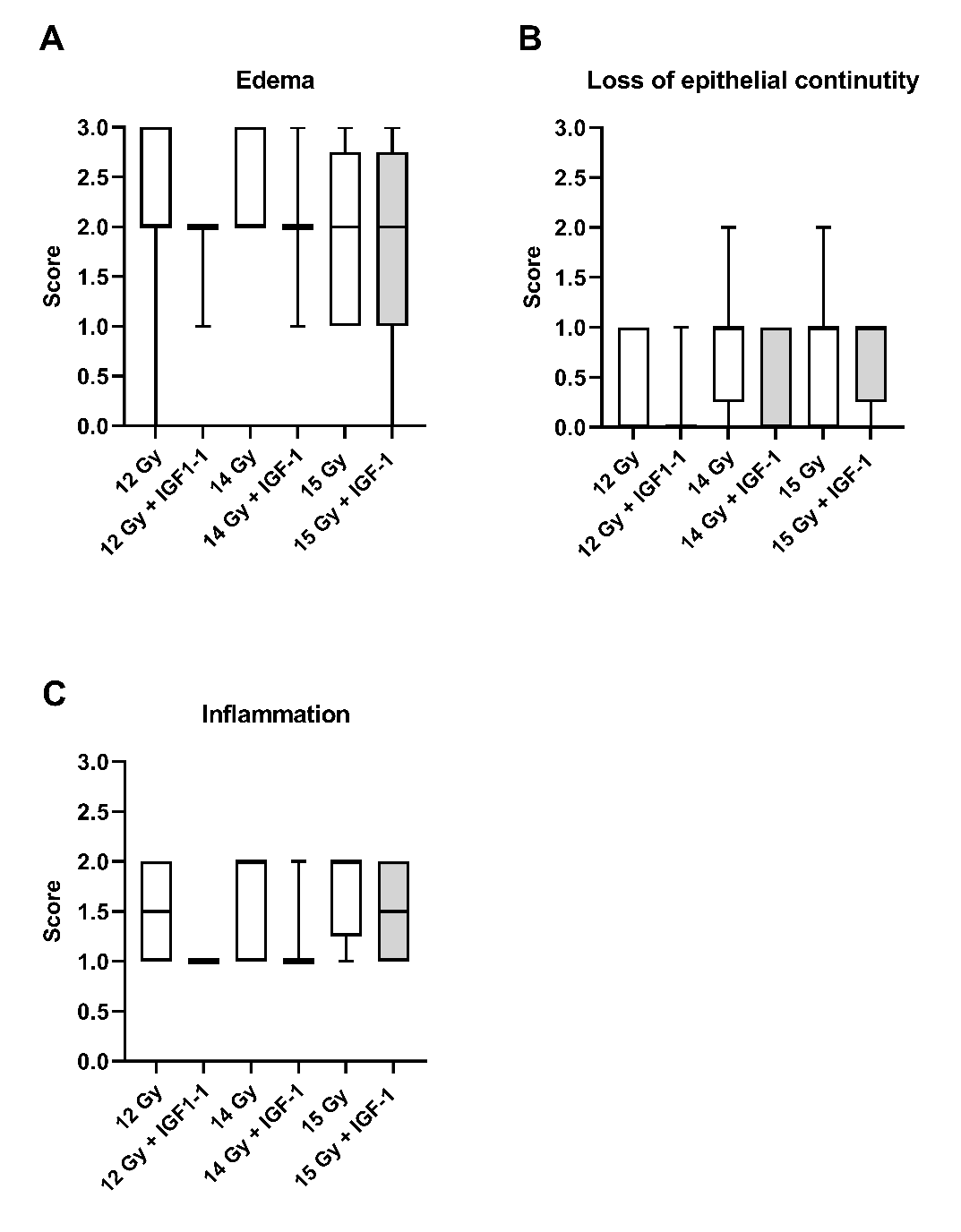
**

**Supplementary Figure 1.** Histopathological changes and effect of different therapeutical regimens of IGF-1 (1 mg/kg) in the duodenum of mice irradiated by 12 and 14 Gy with shielded head and neck. A: edema. B: epithelial integrity. C: inflammation.

**
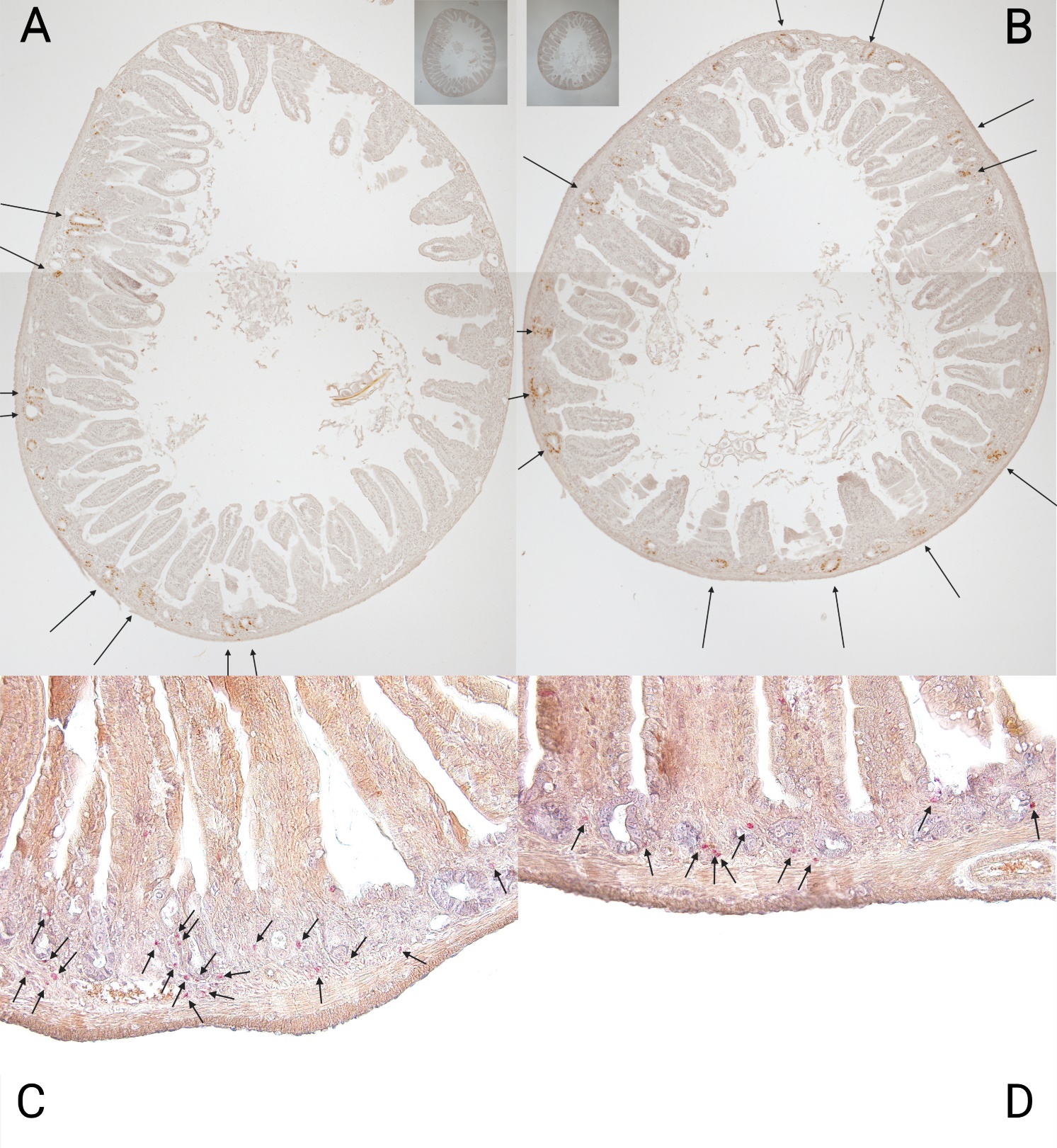
**

**Supplementary Figure 2.** Samples of mouse intestine irradiated by 12 Gy with shielded head and neck were collected 84 h after irradiation. A: non-treated animals (administered with physiological saline). Immunohistochemical detection of BrdU positive cells counterstained with hematoxylin. BrdU was administered intraperitoneally 4 hours before sample collection. Arrows depict surviving crypts with ≥ 10 BrdU positive cells. Due to hardware limitations, the two microphotographs were taken at 40× magnification and merged using BioRender software. The whole sample was also microphotographed at 12.5× magnification but with limited detail resolution (in the upper right corner). B: mice treated with IGF-1 (1 mg/kg) at 1, 24, and 48 h after irradiation. The therapy significantly increased the number of surviving crypts (arrows). C: non-treated animals (administered with physiological saline). Detection of chloroacetate esterase-positive cells (red spots depicted with arrows) counterstained with hematoxylin. Chloroacetate esterase is considered specific for cells of granulocytic lineage. D: IGF-1 therapy reduced the number of infiltrating chloroacetate esterase-positive cells (arrows).


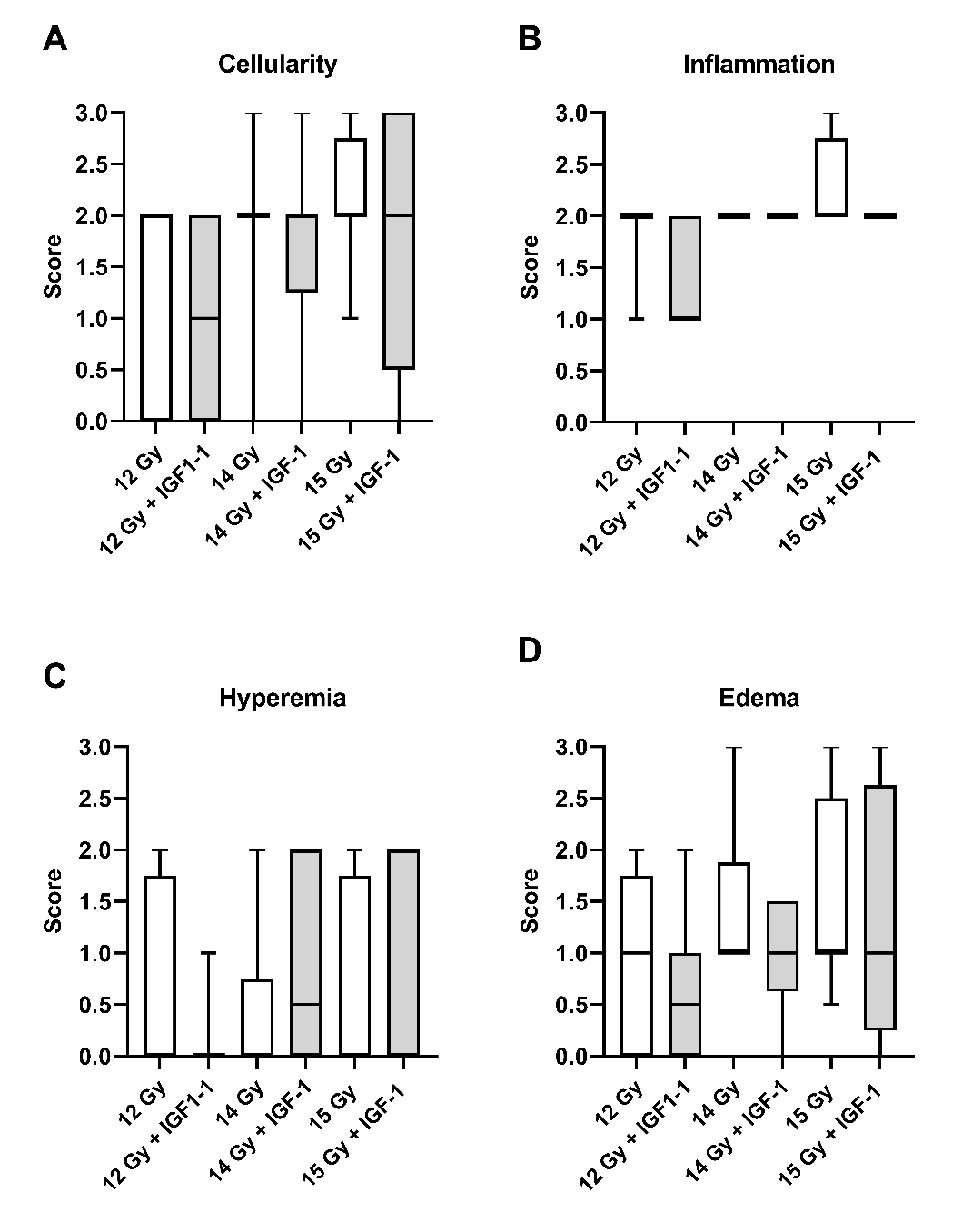


**Supplementary Figure 3**. Histopathological changes and effect of different therapeutical regimens of IGF-1 (1 mg/kg) in the jejunum of mice irradiated by 12 and 14 Gy with shielded head and neck. A: edema. B: epithelial integrity. C: inflammation.

**
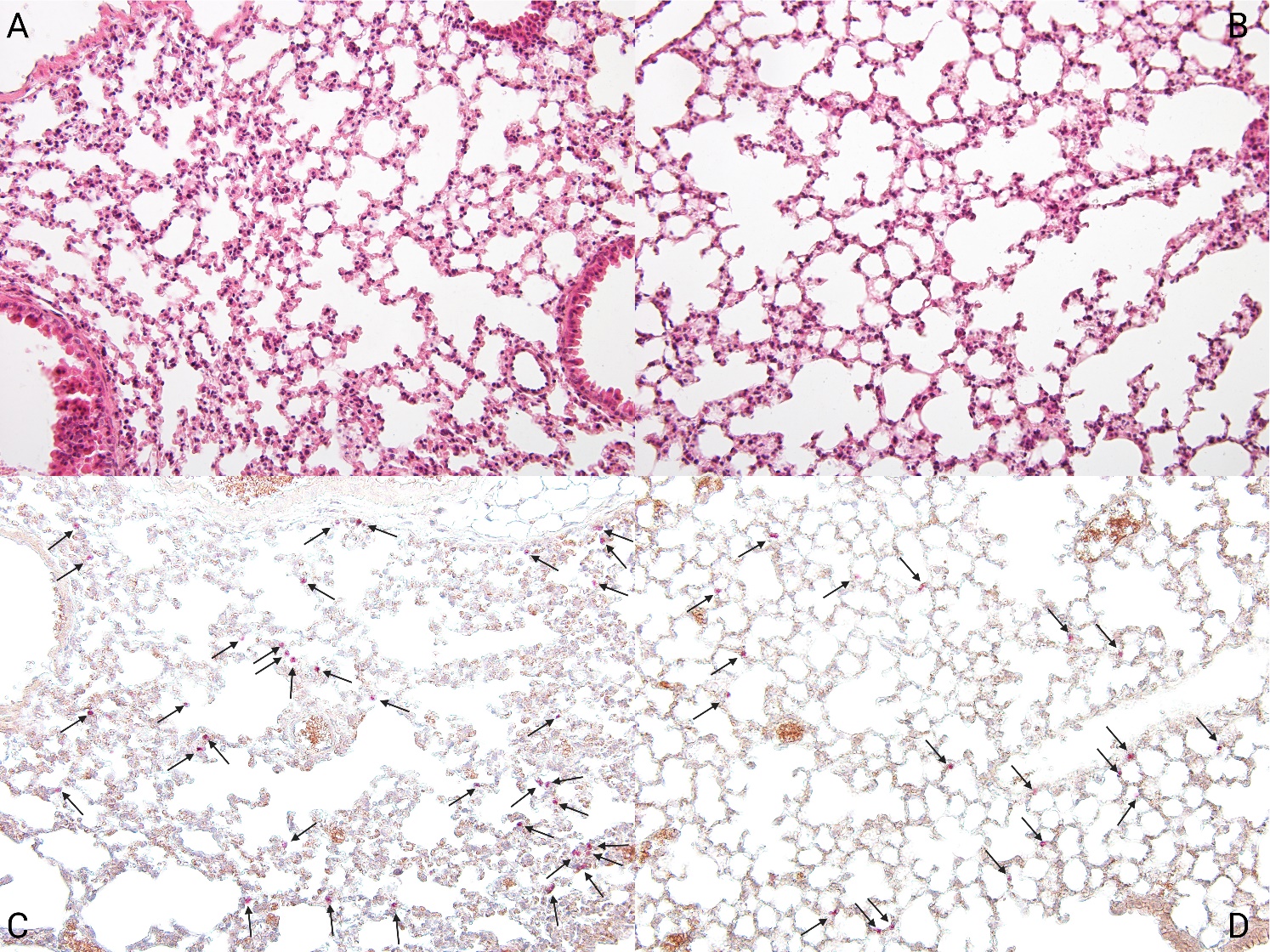
Supplementary Figure 4**. Samples of mouse lungs irradiated by 12 Gy with shielded head and neck were collected 84 h after irradiation. A: non-treated animals (administered with physiological saline). Hematoxylin-eosin-stained sample shows increased cellularity, mild hyperemia, and mild septal edema, reducing the airness of the tissue. B: mice treated with IGF-1 (1 mg/kg) at 1, 24, and 48 h after irradiation. The findings were rather focal, significantly increasing the airness of the tissue. C: non-treated animals (administered with physiological saline). Detection of chloroacetate esterase-positive cells (red spots depicted with arrows) counterstained with hematoxylin. D: IGF-1 therapy reduced the number of infiltrating chloroacetate esterase-positive cells in the lung tissue (arrows).

**
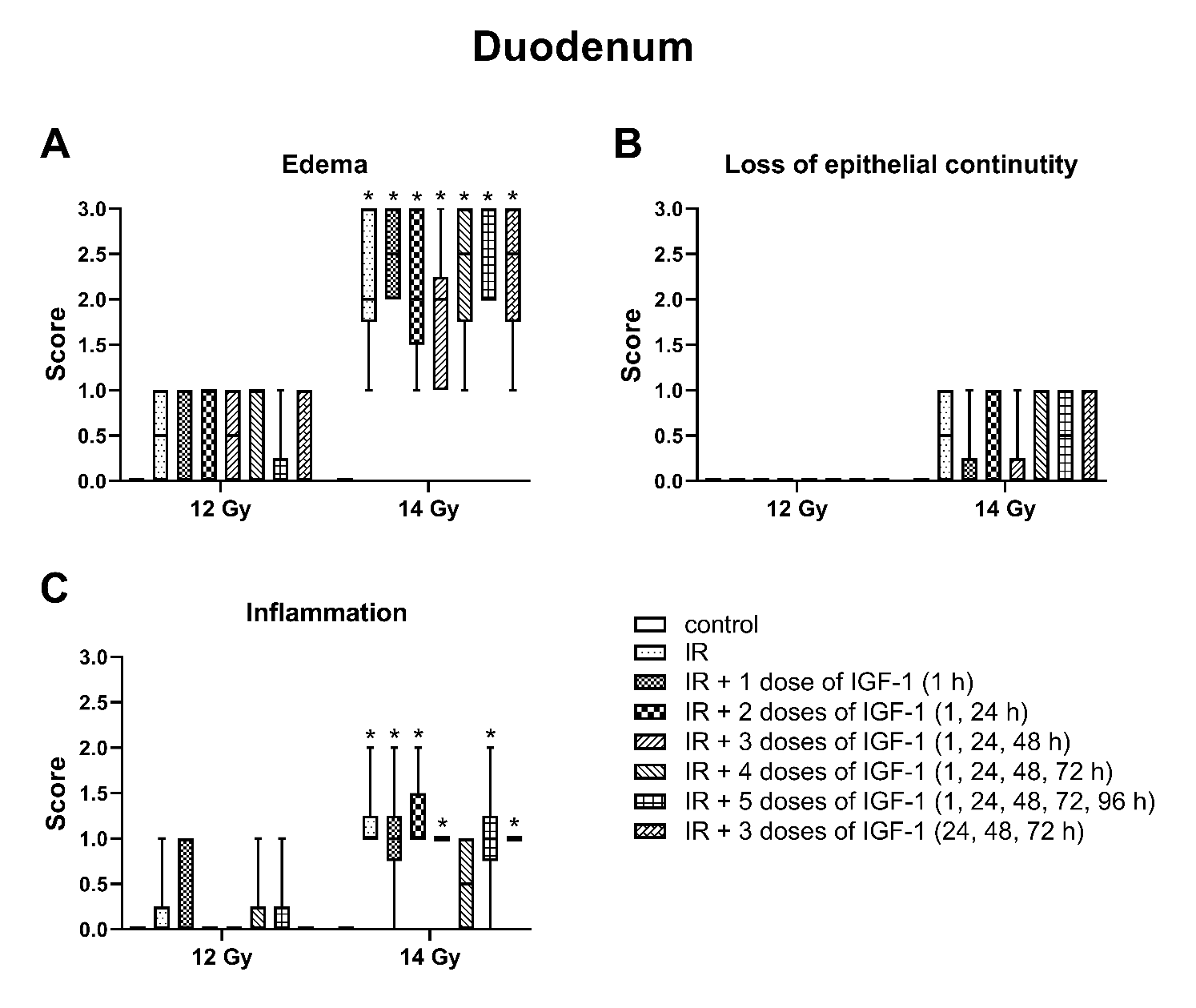
Supplementary Figure 5**. Histopathological changes and effect of different therapeutical regimens of IGF-1 (1 mg/kg) in the ileum of mice irradiated by 12 and 14 Gy with shielded head and neck. A: edema. B: epithelial integrity. C: inflammation.

**
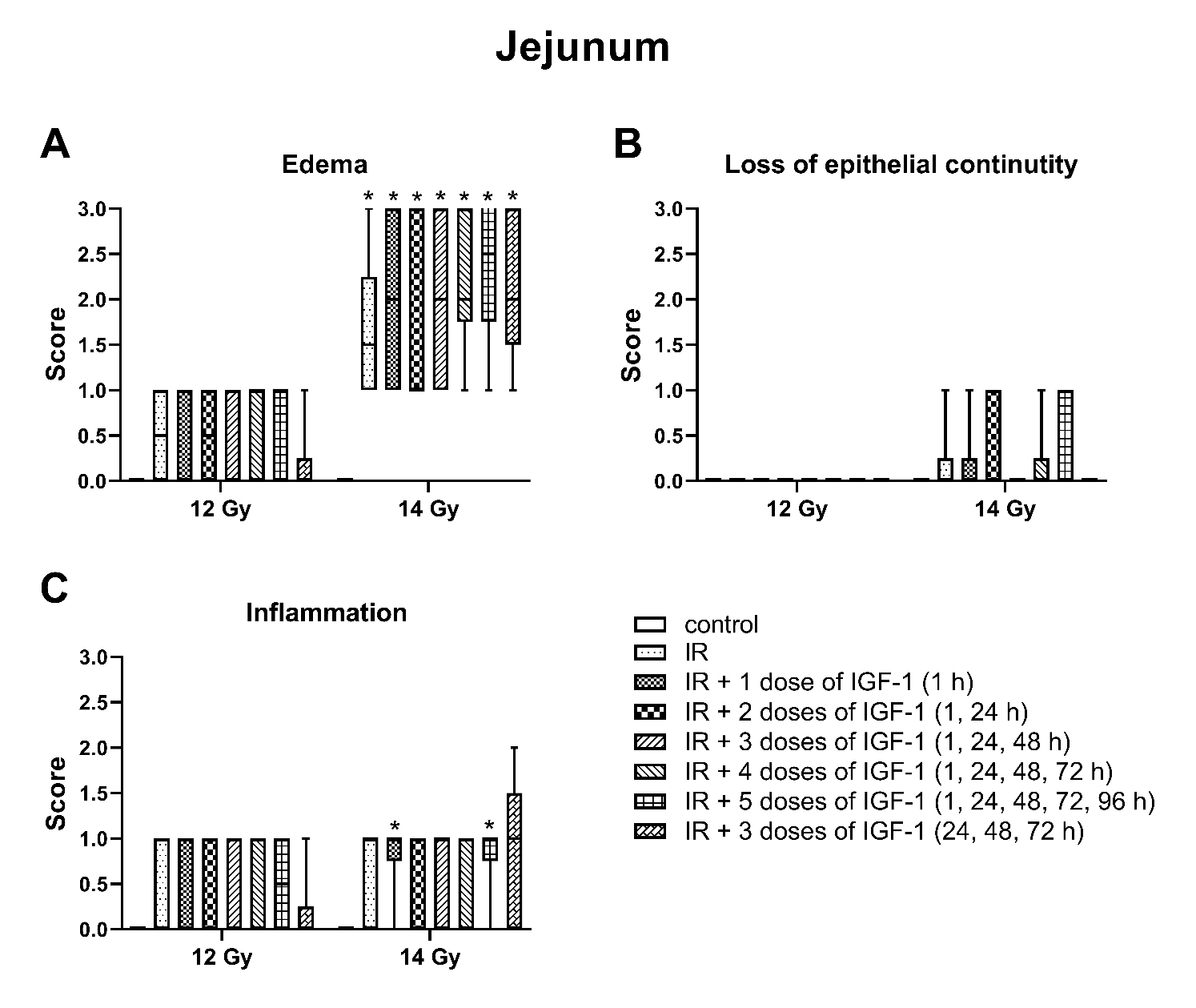
Supplementary Figure 6**. Histopathological changes and effect of different therapeutical regimens of IGF-1 (1 mg/kg) in the jejunum of mice irradiated by 12 and 14 Gy with shielded head and neck. A: edema. B: epithelial integrity. C: inflammation.

**
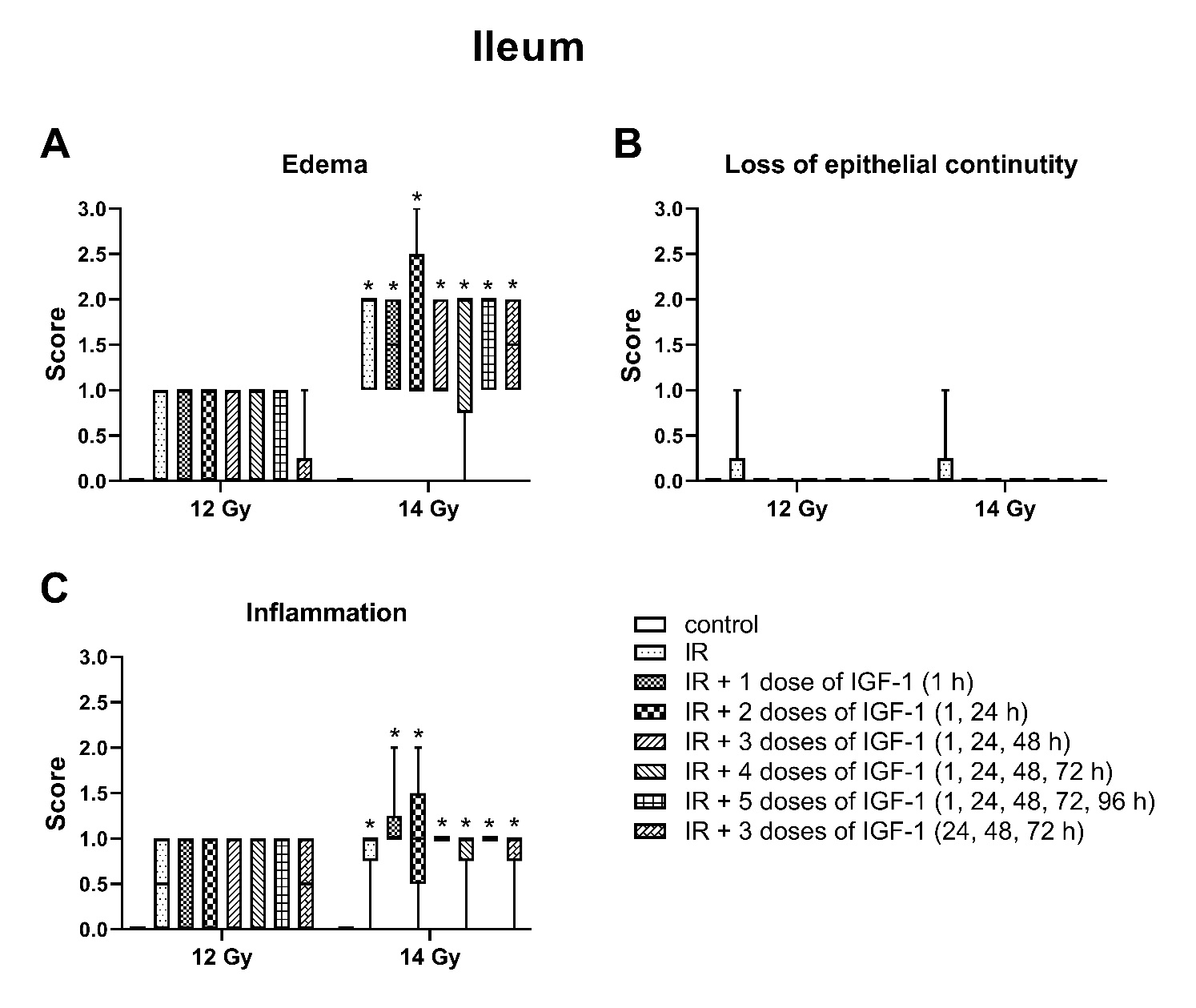
Supplementary Figure 7**. Histopathological changes and effect of different therapeutical regimens of IGF-1 (1 mg/kg) in the ileum of mice irradiated by 12 and 14 Gy with shielded head and neck. A: edema. B: epithelial integrity. C: inflammation.

# Supplementary Tables

**Supplementary Table 1**. Animal survival data.

| Group | Time of death (days after irradiation) |
| --- | --- |
| 14 Gy | 7, 7, 126, 134, 139, 153, 159, 160, 164, 164, 166, 167, 181, 181, 188, 202, 206, 206, 207, 209 |
| 14 Gy + 1.0 mg/kg IGF-1 | 127, 129, 134, 146, 164, 168, 168, 169, 171, 176, 181, 191, 197, 205, 208, 219, 223, 249, 255, 292 |
| 14.5 Gy | 5, 6, 7, 7, 8, 122, 126, 139, 148, 148, 148, 150, 153, 153, 160, 181, 183, 217, 217, 236 |
| 14.5 Gy + 1.0 mg/kg IGF-1 | 6, 6, 6, 7, 7, 7, 7, 8, 130, 131, 132, 139, 141, 144, 145, 153, 153, 155, 233, 263 |
| 15 Gy | 5, 6, 6, 6, 6, 6, 6, 6, 6, 7, 7, 7, 7, 149, 157, 161, 164, 168, 173, 175 |
| 15 Gy + 1.0 mg/kg IGF-1 | 6, 6, 6, 6, 6, 6, 6, 6, 6, 7, 7, 7, 8, 8, 9, 148, 158, 158, 159, 170 |
